# Occupancy of the Ethiopian endemic Moorland Francolin in pristine and degraded Afroalpine biome using a camera trap approach

**DOI:** 10.1002/ece3.10551

**Published:** 2023-10-31

**Authors:** Abadi Mehari Abrha, Kai Gedeon, Lars Podsiadlowski, Demis Mamo Weldesilasie, Till Töpfer

**Affiliations:** ^1^ Leibniz Institute for the Analysis of Biodiversity Change Bonn Germany; ^2^ Institute for Evolutionary Biology and Ecology University of Bonn Bonn Germany; ^3^ Department of Animal, Rangeland and Wildlife Science Mekelle University Mekelle Ethiopia; ^4^ Department of Wildlife and Ecotourism Management Guassa Community Conservation Area Mehal Meda Ethiopia

**Keywords:** Afroalpine biome, camera trap, conservation, endemic, moorland francolin, occupancy

## Abstract

Occupancy modeling is an essential tool for understanding species‐habitat associations, thereby helping to plan the conservation of rare and threatened wildlife species. The conservation status and ecology of several avian species, particularly ground‐dwelling birds, are poorly known in Ethiopia. We used camera trap‐based occupancy modeling to investigate habitat covariate influence on occupancy (Ψ) and detection probability (ρ) estimates of Moorland Francolins *Scleroptila psilolaema* from spatially replicated surveys across both relatively pristine and disturbed landscapes in the Afroalpine biome of Ethiopia. Model‐averaged estimate of $\hat{\psi }$ across all sites was 0.76 (SD = 0.28) and $\hat{\rho }$ was 0.77 (SD = 0.13) in the pristine landscape. The $\hat{\psi }$ of the species in the disturbed landscape was 0.56 (SD = 0.19) and $\hat{\rho }$ was 0.48 (SD = 0.06). As hypothesized, based on our model‐averaged beta coefficient estimates (β_mean_ ± SE), predators significantly negatively influenced the occupancy of Moorland Francolins in pristine habitat. We also found a significant positive association of occupancy with herb species richness. Contrary to our prediction, distance to road significantly negatively influence the occupancy of the species, suggesting that occupancy probability was highest in proximity to roadsides and trails in the pristine habitat. There was no significant influence of habitat covariates on the occupancy of the species in the disturbed habitat. The most important covariates that significantly influence the detectability of the species in pristine habitat included sampling occasion and precipitation. The greater occupancy and detectability of this endemic species in the pristine habitat could be linked with the particular conservation status and management of this biodiversity hotspot in the central highlands of Ethiopia. Our results suggest that strict legal enforcement is required to sustainably preserve Moorland Francolins and the ecological integrity of the entire Afroalpine biome. We recommend using camera traps in order to develop realistic and effective conservation and management strategies for rare, sensitive, cryptic, and ground‐dwelling animals in the region.

## Graphical abstract

In this research, camera trap‐based occupancy modeling was applied to investigate habitat covariates' influence on occupancy and detection probability estimates of Moorland Francolins from spatially replicated surveys in relatively pristine and disturbed landscapes in the Afroalpine biome of Ethiopia. The study species was influenced by predators, herb species richness, and distance to roads and trails in the pristine habitat. Camera traps help to plan effective conservation and management strategies for secretive ground‐dwelling animals in the topographically complex region.

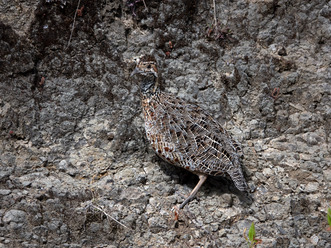

## 1 INTRODUCTION

Among the 34 Earth's biodiversity hotspots, the Eastern Afromontane hotspot, including the Ethiopian Highlands, ranks fourth by a number of endemic plant and vertebrate families and genera (Mittermeier et al., 2004). Next to the Guinea‐Congo Forests biome, the second‐highest number of biome‐confined bird species are found in the Afrotropical Highlands biome (BirdLife International, 2004). In Ethiopia, all bird species subsist in three biomes: the Afrotropical Highlands (including the Afroalpine and Afromontane), Sudan‐Guinea savanna, and Somali‐Masai biomes (Fishpool & Evans, 2001; Gedeon, Zewdie, & Töpfer, 2017). The Afroalpine biome of Ethiopia consists of a complex mosaic of grassland, moorland, bushland, and other habitat types which are unique in terms of species distinctiveness. This biome harbors a considerable endemic flora and fauna and is home to a number of range‐restricted bird species (Ash & Atkins, 2009; Gedeon, Zewdie, & Töpfer, 2017; Töpfer & Gedeon, 2020), as well as to rodents (Ashenafi et al., 2012; Bryja et al., 2019; Razgour et al., 2021), and medium and large‐sized mammals (Ashenafi & Leader‐Williams, 2005).

Historically, the oldest records of human high‐elevational occupation worldwide are from the Afroalpine biome (Ossendorf et al., 2019), but today human population growth (Reber et al., 2018) is the key threat to wildlife in the Afroalpine and Afromontane (Asefa et al., 2017; Ashenafi et al., 2012; Razgour et al., 2021). Agricultural practices, human‐induced climate change, and other threats synergistically affect both the biomes' flora (Asefa et al., 2020) and fauna (Asefa et al., 2017; Razgour et al., 2021; Rodrigues et al., 2021).

Like in other tropical countries, the distribution of vegetation in Ethiopia reflects the interplay among altitudinal variation as well as climatic and other abiotic factors (Friis et al., 2010). The combination of different habitat characteristics, species traits, and their interactions define the occurrence, occupancy, and abundance of wildlife populations and influence their distribution patterns and detectability (Devarajan et al., 2020; Guillera‐Arroita, 2017).

**FIGURE 1:**
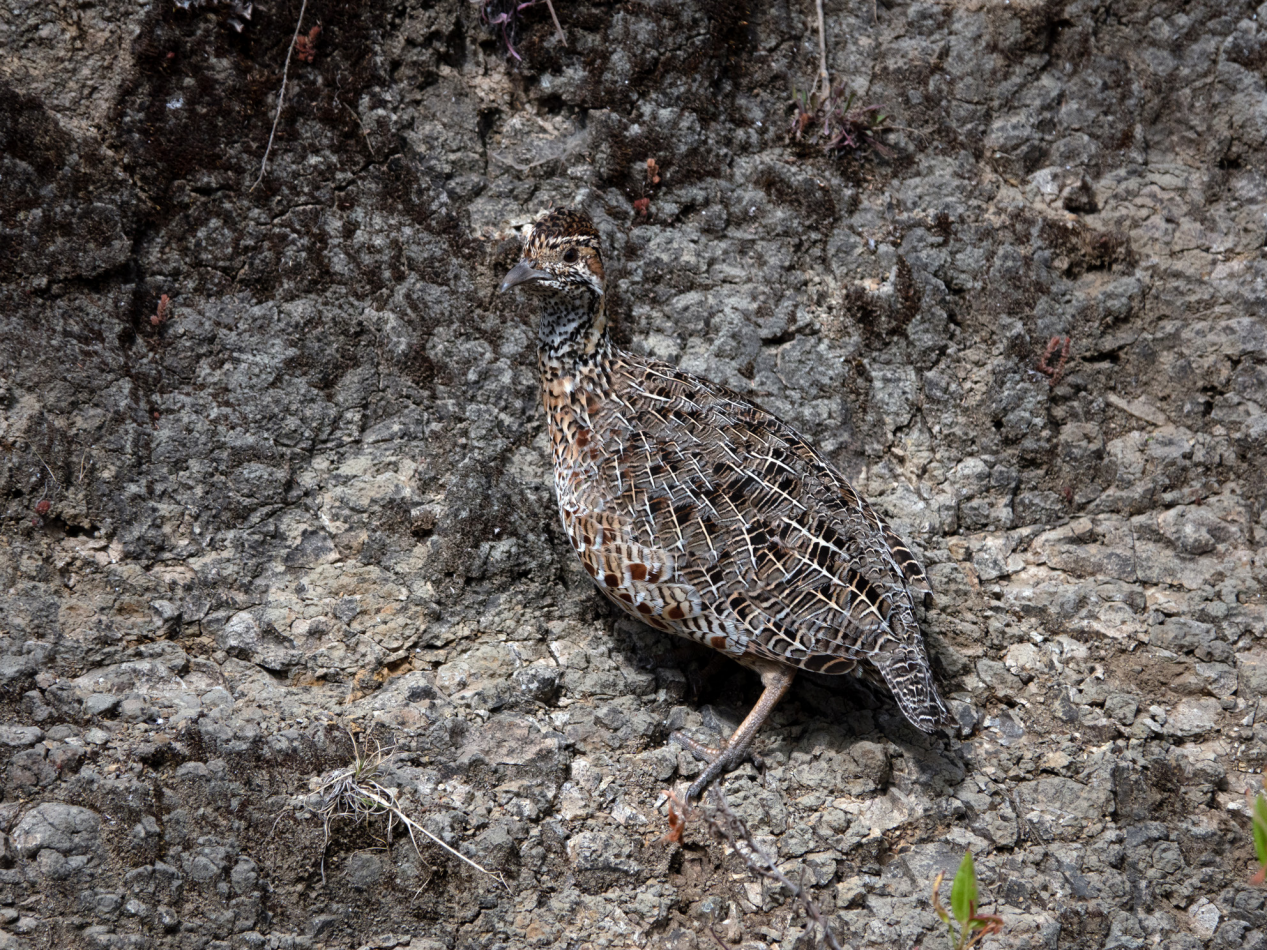
An adult female Moorland Francolin *Scleroptila psilolaema* in the Afroalpine biome, Ethiopia. The feather patterns contribute crypsis through background matching in this species (photo credit: Kai Gedeon).

Most native bird species of Afroalpine and Afromontane habitats of Ethiopia are poorly studied in terms of their abundance, distribution, and threats (Ash & Atkins, 2009; Gedeon, Zewdie, & Töpfer, 2017). One of them is the Moorland Francolin *Scleroptila psilolaema* (Figure 1), an endemic species of the Ethiopian highlands (BirdLife International, 2023; Gill et al., 2023), where it inhabits both Afroalpine and Afromontane habitats (Töpfer & Gedeon, 2020). Knowledge on its breeding biology, home range size, population abundance, occupancy (i.e., habitat use), and other ecological patterns is still scant. Previous distributional data showed Moorland Francolins to occur in the eastern and western highlands (Ash & Atkins, 2009; Gedeon, Zewdie, & Töpfer, 2017). It is classified as Near Threatened due to the ever‐increasing loss of moorland and grassland habitats (BirdLife International, 2023), but its population size and habitat association along its geographical range are insufficiently known.

In biodiversity‐rich Sub‐Saharan African countries such as Ethiopia, little attention is paid to camera trap‐based research (Cordier et al., 2022). To fill this knowledge gap, our sampling protocol for Moorland Francolins occupancy estimates relies on data obtained using camera traps. Although this approach may disturb wildlife and alter their behavior (Caravaggi et al., 2020; Wearn & Glover‐Kapfer, 2017, 2019), it is cost‐effective and non‐invasive to study ecological patterns such as population size and distribution of animals. The centerpiece in most occupancy‐based camera trap studies are frequently applied on mammal species (e.g., Burton et al., 2015; Niedballa et al., 2015; Kays et al., 2020; Cremonesi et al., 2021; Wevers et al., 2021; Cordier et al., 2022), yet some studies are conducted on ground‐dwelling bird species, mainly pheasants (e.g., O'Brien & Kinnaird, 2008; Sharief et al., 2022; Tanwar et al., 2021; Zou et al., 2019). Most importantly, camera traps are particularly useful to study elusive, cryptic, and rare species (O'Brien & Kinnaird, 2008; Sharief et al., 2022; Si et al., 2014; Winarni et al., 2005) and thus represent the most promising approach to investigate Moorland Francolin. Camera trapping is more efficient than other methods such as traditional distance sampling (Suwanrat et al., 2015; Wearn & Glover‐Kapfer, 2019). Moreover, it can provide valuable information to implement sound conservation strategies (O'Brien & Kinnaird, 2008; Sharief et al., 2022; Si et al., 2014; Wearn & Glover‐Kapfer, 2017).

We attempt to draw an inference of baseline data on the ecology of Moorland Francolins using an occupancy modeling framework. We used presence/absence (i.e., detection/non‐detection) data to analyze two stochastic processes: occupancy and detection probability. Occupancy is a dichotomous state variable that accounts for imperfect detection to minimize unreliable inferences of species distribution and range (Bailey et al., 2014; Guillera‐Arroita & Lahoz‐Monfort, 2012; Kéry et al., 2010; MacKenzie et al., 2018; Tyre et al., 2003). Occupancy models estimate the probability of a species' presence in a fraction of landscape units (MacKenzie et al., 2002, 2018) and help to understand habitat use within a landscape. They are applied across several animal taxa for the implementation of successful conservation and management strategies (Burton et al., 2015; MacKenzie et al., 2018; Steenweg et al., 2017). Therefore, the objective of this study was to gain insight into the habitat use of Moorland Francolins in its native range for the first time and to investigate the effect of habitat covariates on occupancy and detection probability from spatially replicated surveys.

## 2 MATERIALS AND METHODS

### 2.1 Study area

**FIGURE 2:**
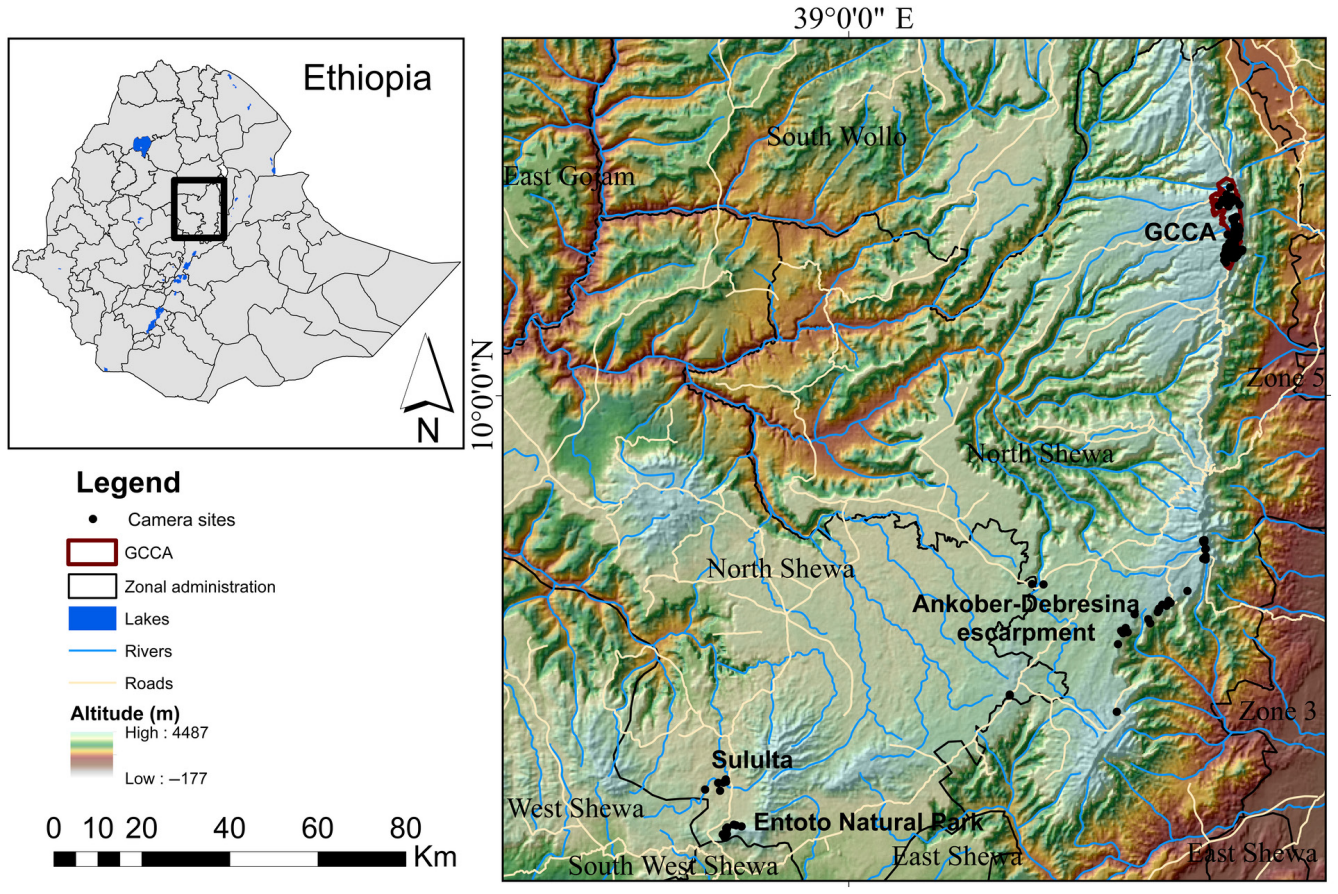
The two study areas (GCCA and SEA) and location of camera sites in the central highlands of Ethiopia. GCCA, Guassa Community Conservation Area. The southern sites (including Sululta, Entoto National Park, Ankober‐Debresina escarpment, and other areas) form SEA.

This study was performed in two areas (Figure 2): Guassa Community Conservation Area (hereafter GCCA) and an area encompassing Sululta plain, Entoto Natural Park, Ankober‐Debresina escarpment, and a few sites between them (hereafter collectively abbreviated SEA). The study areas are part of Ethiopia's central highlands in which several Important Bird and Biodiversity Areas (IBAs) are designated (Tilahun et al., 1996). These highland areas consist of top mountain massifs and volcanic cones (Friis et al., 2010). Most of our study sites (93%) were located in IBAs, including GCCA, Entoto Natural Park, Ankober‐Debresina escarpment, and Sululta plain. The remaining sites were located outside these IBAs in Angolela Tera, Assagirt, Sheno, and Mendida districts. However, both IBAs and non‐IBAs sites in SEA are under serious anthropogenic threat: farming, livestock grazing, settlement, monocultural plantations, and recreational activities. For instance, ENP has shifted its purpose from conservation implementation (Tilahun et al., 1996) to recreational area where mass tourism (Asefa, 2018; Tesema & Berhan, 2019) and monocultural plantations (Bahru et al., 2021; Tadesse & Tafere, 2017) strongly affect the landscape. Both Sululta plain and Ankober‐Debresina escarpment are mainly influenced by livestock grazing, farming, and settlement expansions. Except for Sululta plain, the other areas are dominated by exotic *Eucalyptus* plantation and African juniper *Juniperus procera* (Esayas & Bekele, 2011). Therefore, we distinguished between the two study areas based on their different levels of human disturbance, topography, floristic structure and composition, and conservation status, considering GCCA a relatively pristine and SEA a strongly human‐modified area.

**FIGURE 3:**
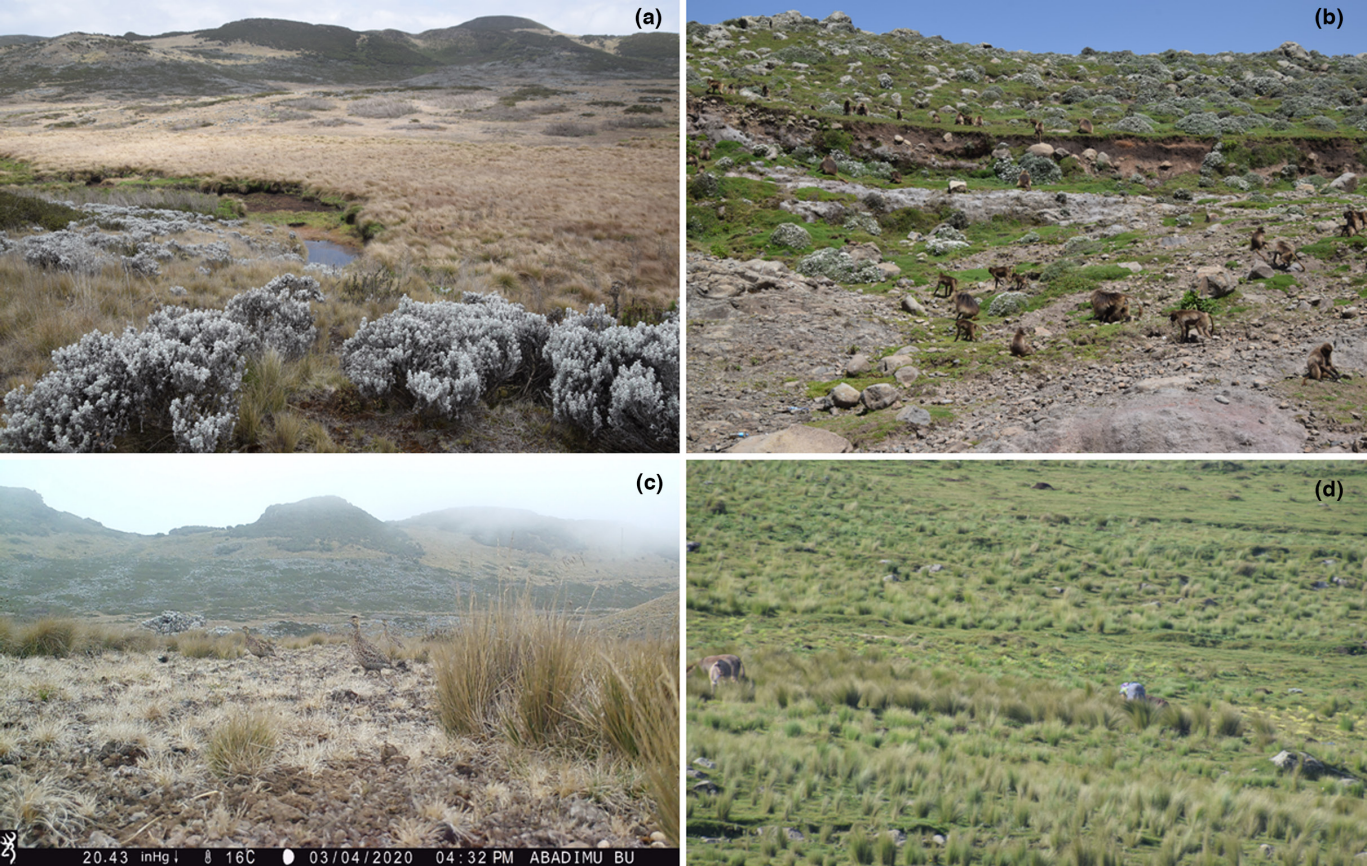
Afroalpine habitats in the central highlands of Ethiopia: GCCA with most habitat types (a) and the target species feeding in *Helichrysum‐Festuca* grassland (c) and SEA with degraded rocky habitat (b) and grazing land (d).

GCCA (Figures 2 and 3) covers 78 km^2^ (Steger et al., 2020), yet the total land area sums up to 111 km^2^ if the adjoining villages and other land use types are included (Ashenafi & Leader‐Williams, 2005; Nigussie et al., 2019). This area shows critically important habitat features for many wildlife species (Steger et al., 2020) and comprises both the Ericaceous belt (3000–3200 m a.s.l) and the Afroalpine belt (above 3200 m a.s.l) (Friis et al., 2010). The area has been managed by the local community through a management model called the Qero system (Ashenafi et al., 2012; Ashenafi & Leader‐Williams, 2005). Unlike other IBAs of the study areas, the Qero system, coupled with the conservation initiatives of Frankfurt Zoological Society, The Darwin Initiative, European Union, and Ethiopian Wolf Conservation Program have significantly sustained the ecological integrity of GCCA since 2003. In this area, the Ethiopian Wolf *Canis simensis* is the flagship species (Tefera & Sillero‐Zubiri, 2006), generating income through ecotourism which is partly plowed back for the conservation of the species itself (Eshete et al., 2015; Estifanos et al., 2018).

SEA (Figures 2 and 3) forms part of the Afromontane with altitudes generally below 3000 m a.s.l. Very small patches of herbs, shrubs, scattered acacia trees, and exotic trees are common. Here, the Moorland Francolins persist in very small uncultivated and grassland patches of Afromontane habitats.

These highland areas experience a bimodal rainfall pattern with main rain from June to September and smaller amount of rain from October to February (Mohammed et al., 2022). The distinctive habitat features of both of these areas are erratic climatic conditions and a very short dry season (ca. 2 months). The mean annual temperature of GCCA and SEA are 21.26°C (± 0.95 SE) and 15.53°C (± 0.55 SE), whereas the mean annual precipitation of GCCA and SEA were 2.65 mm (± 0.78 SD) and 2.69 mm (± 0.90 SD), respectively (Figure S1).

### 2.2 Sampling design

Site selection for this study was made randomly. Most sites were obtained through a distribution map from the IUCN, scientific literature, and citizen science data, whereas some sites were chosen without antecedent species records. Following the standard design procedure for allocating optimal sampling occasion (MacKenzie et al., 2018; MacKenzie & Royle, 2005), we initially attempted to conduct a total of 185 camera sites (or preferably sites) (*n* = 116 for GCCA and *n* = 69 for SEA) for a single‐season design located in various habitat types. All camera sites were arranged in 39 line transects (*n* = 20 for GGCA and *n* = 19 for SEA), with an average transect length of 2.04 km (± 0.80 SD) across both study areas. In this study, we expected that the number of sites (*s*) and occasions (K) were sufficient to determine the stochastic processes. Then, the total survey is simply defined as *s ×* K, and the maximum survey occasion for each site was calculated by minimizing *s*, while taking a standard error of 0.05 for GCCA and 0.065 for SEA. Since both study areas are separated by approximately 150 km independent camera trap data collections were conducted for 5 months for both areas. Along these geographical scales, specific habitat characteristics (i.e., covariates) predicted to influence the occupancy and detection rates of the target species were measured at each site (Table 1).

**TABLE 1:** Habitat covariates predicted to affect occupancy and detection probabilities of Moorland Francolins in the central highlands of Ethiopia.

Covariate	Type of data	Measurement and scoring systems	Hypothesized relationship	References tested the effects
Occupancy covariates				
Fine‐scale level covariates				
Herb species richness (Hsp)	Continuous	Number of herb species in each site	The species prefers herbaceous sites for feeding, breeding, and concealment.	Jolli et al. (2012); Sukumal et al. (2017)
Species richness (Sprich)^a^	Continuous	Plant species richness (i.e., alpha diversity) in each site	See Hsp	Atikah et al. (2021)
Woody density (WD)	Continuous	Density of tree and shrub vegetation per 0.8 ha	The species is negatively influenced by birds of prey perched on trees and rocky areas.	Sukumal et al. (2017)
Tree canopy cover (T_caco_)	Continuous	Tree canopy cover (CaCo) index estimated using mobile app or digital camera	Francolins avoid tree canopy cover due to the presence of human disturbance and birds of prey and other predators.	Atikah et al. (2021); Chen et al. (2019); Sukumal et al. (2017)
Predator^b^	Binary	Presence of predator (1 = if predator/s was/were recorded and 0 otherwise).	Francolin are negatively influenced by predators.	Abrha et al. (2018); Sukumal et al. (2017)
Landscape‐scale covariates				
Elevation (Elev)	Continuous	The elevation of each site is measured in the field using GPS.	Elevation explains climate and vegetation variations that affect species survival and reproduction differently in both sites.	Chen et al. (2019); Holzner et al. (2021); Jolli et al. (2012); O'Brien and Kinnaird (2008); Pardo et al. (2017); Wevers et al. (2021); Whitworth et al. (2018)
Distance to roads (DR)^c^	Continuous	Distance from the center of each site to the nearest paved or unpaved roads	Proximity to road exposes the species to predators and other disturbances.	Dean et al. (2019); Kroeger et al. (2022); Semper‐Pascual et al. (2020); Tan et al., (2017); Whitworth et al. (2018)
Distance to settlements (DS)^c^	Continuous	Distance from the center of each site to the nearest settlement	Francolins avoid human settlements where several stressors, including human presence, grazing, mowing, and others are common activities.	Chen et al. (2019); Jolli et al. (2012); Nuttall et al. (2017); O'Brien and Kinnaird (2008); Pardo et al. (2017); Semper‐Pascual et al. (2020)
Distance to water point (DW)	Continuous	Distance from the center of each site to the nearest water point (wetlands, streams, madicolous, etc)	Francolins use water points for food and cover in various habitats	Nuttall et al. (2017); Sukumal et al. (2017)
Detection covariates				
Fine‐scale level covariates				
Sampling month (M)^a^	Continuous	The survey month for both areas (SEA: Feb and Mar and GCCA: Apr‐Jun) in 2020	Detection probability of Moorland Francolins varies between sampling months	Holzner et al. (2021); Jolli et al. (2012)
Survey occasion (E)	Continuous	Number of days for which camera trap was active in each site per sampling occasion also called survey timing‐how long?	The francolin detection increases with number of days of cameras deployed	Holzner et al. (2021); Kays et al. (2020); Semper‐Pascual et al. (2020); Si et al. (2014); Tan et al., (2017); Wevers et al. (2021)
Climate covariates				
Temperature (T)	Continuous	Temperature of each site while camera trap was active	The francolin detection is influenced by temperature because francolins highly favor cold conditions and adapted to extreme low nocturnal temperature	Abrha et al. (2018); Gedeon, Rödder, et al. (2017)
Precipitation (P)	Continuous	Precipitation of each site while camera trap was active	Francolins have plenty of food resource to easily rake the wet ground and produce continuous calls for breeding during raining or wet season	Abrha et al. (2018); Gedeon, Rödder, et al. (2017)

*Note*: The first nine predictors are site‐specific covariates, whereas the last four are observational‐specific covariates.

^a^ The spatiotemporal covariates are dropped due to high collinearity (Dormann et al., 2013; Zuur et al., 2010). This study selected herb species richness over total species richness (Sprich) in both study area. Herbaceous and shrubby vegetation were dominant in GCCA (> 80% ground vegetation cover) (Nigussie et al., 2019).

^b^ Hunting was not considered as a threat for this species (See Discussion).

^c^ Human disturbance factors: grazing, mowing and farming are the major factors in the study sites (Ashenafi et al., 2012; Nigussie et al., 2019; Steger et al., 2020). *Festuca abyssinica* grass (Guassa) intriguingly is valued for fodder for livestock (cut and carrying system and livestock grazing), thatching, wall building mix with mud, and help to make whip, rope, hat, broom (mure) and raincoats (gesa).

### 2.3 Camera trapping

In December 2019 and the first 3 weeks of January 2020, we made a pilot survey in both areas to assess the study species using camera traps and broadcast playback methods. A total of 20 cameras (Browning Trail Cameras and Bushnell Trophy Cam HD brands) were used for short‐term deployments in this study. Since we had a small number of cameras, some adjoining habitats (see habitat covariates below) were simultaneously assessed and in both study areas cameras were deployed sequentially. Cameras were repositioned to other sites to cover the desired representative home range and to make the field survey more cost‐effective. When small camera traps are available, repositioning to new sites is recommended to increase the spatial coverage of target species (Meek et al., 2014; Shannon et al., 2014; Si et al., 2014; Wearn & Glover‐Kapfer, 2017).

Each camera trap was placed horizontally (i.e., camera alignment was perpendicular to the ground) within a 50 m radius (~ 0.8 ha) of plot or focal patch size to optimize detectability. Because some terrain settings were very difficult to conduct surveys, cameras were not fixed at the center of each plot instead they were placed approximately 10–30 m distance from the grid center, where freshly raked and possible feeding grounds were noticed. Single camera placement is employed to detect small‐medium mammals and bird species (Ferreguetti et al., 2015; Lamelas‐López & Salgado, 2021). The camera spacing in continuous habitats in GCCA was approximately 0.3 km (0.2–0.5 km), while in SEA was approximately 0.5 km (0.3–0.8 km) to enhance detectability and to avoid spatial autocorrelation between camera traps. Though telemetry data collection was originally proposed to estimate the home range of the species which enables to estimate camera spacing, we assumed that the camera trapping space was sufficient and representative to study occupancy of this species based on available literature. If the average home range size of a target species is not known, it is recommended to infer spatial extent from congeneric or other related species (Niedballa et al., 2015). Mostly, camera trap spacing, based on home range, for pheasants ranges from 0.2 km (Zou et al., 2019) to 0.7 km (Suwanrat et al., 2015). Therefore, the camera spacing was higher than the home range diameter of the species, which was a similar approach as in other studies (Maffei & Noss, 2008; Niedballa et al., 2015). In our case, camera traps were unbaited but rather were providentially camouflaged with rocks, stones, and Ericaceous heathlands of the study sites. Site selection for camera placement was randomly carried out across various habitats of both study areas, as was proposed by several other studies (e.g., Burton et al., 2015; Cordier et al., 2022; Meek et al., 2014; Tanwar et al., 2021; Wearn & Glover‐Kapfer, 2017).

We placed camera traps on tree trunks, attached to thick coarser grasses (*Festuca* spp.) and shrubs, and on wooden stakes at approximately 30–60 cm above the ground, as this standard height is credible to trigger the motion sensor and it is reasonable to detect ground‐dwelling bird species (Figure 3; Figure S2). Because some sites were in completely rocky areas, we also put cameras by arranging stacked stones that matched the background of the site. Most cameras had 16 GB memory and some cameras mounted on courser grasses and shrubs had 32 GB SanDisk memory card as they were easily triggered by the movement of vegetation during high wind velocity. However, to enhance good photographs and detectability, prudent vegetation removal was carried out in some sites to avoid false triggering mainly during windy conditions (Meek et al., 2014; Wearn & Glover‐Kapfer, 2017). Our primary interest was to capture photos of the target species that can be easily pooled into detection/non‐detection binary matrices. In most cases, the video function was discounted, yet some videos were collected from the field to understand the natural behavioral repertoire of the species and its interaction with other species (i.e., predators) in the habitats. Because both camera models had different setting options but similar functions, we set up cameras for the following typical important parameters: (1) camera traps were active for 24 h/day and programmed to capture 1 photo/trigger at 10 s intervals, and some sites with more than one camera traps set to capture 20 s video/trigger, with subsequent videos delayed for 5 min; (2) the sensitivity of the infrared sensor was programmed to be medium or normal; and (3) the quality of photos were adjusted to be medium for both camera brands. The battery life of each camera was checked during data retrieval, storage, and repositioning of cameras. Extreme weather conditions (too hot or too cold) severely affected the sensitivity of sensors in our areas.

### 2.4 Habitat covariates

To include representative habitat types in GCCA, we adapted the habitat classifications of Ashenafi et al. (2012). The habitat types were Mima Mound, *Erica* Moorland, *Euryops*‐*Alchemilla* shrubland, *Helichrysum*‐*Festuca* grassland, and *Festuca* (Guassa) Grassland. In their classifications, swamp habitat which is typically characterized by woody vegetation (US definition) and reed swamp or forested fen (European definition) is now replaced by “peatland”. In this habitat, the wetland type is normally a moor surrounded by *Erica*, *Festuca* and other plant species and has permanent and ephemeral water fed by precipitation hence called “ombrothropic peatland”. Moreover, we identified and added montane forest to the classification as an important other habitat type for wildlife species in the area, though it was not included in the rodent‐based study (Ashenafi et al., 2012). Because the sites in SEA study area were human‐dominated, the habitat types were homogenous and it was very hard to distinguish and classify in relation to vegetation patterns. Broadly, we categorized the habitats into *Eucalyptus*‐*Juniperus* habitat and grazing lands. The later class obviously incorporated agricultural lands. Overall, this area has been heavily transformed to *Eucalyptus* plantations to meet demand for wood products and improve the livelihoods of local communities (Bahru et al., 2021; Tadesse & Tafere, 2017).

At the sites, we collected 13 covariates derived from habitat features, landscape connectivity metrics, climatic factors, and sampling covariates which were predicted to influence the occupancy and detection probabilities of the target species. Occupancy was modeled as a function of site‐specific covariates, including biotic factors (vegetation traits and predators) and landscape connectivity metrics, while detectability was modeled as a function of observational‐specific covariates, including survey occasion (hereafter occasion) and climatic factors (precipitation and temperature). The occasion is defined as a total number of days for which each camera was active per site (Table 1).

Specific vegetation traits assumed to influence habitat use were collected from each site using different tools. Due to the occurrence of scattered trees within most sites (with the exception of montane forest adjoining to the moorland habitats and ENP) and complex landscapes varying with soil, climate, topographic, and other features, we used only two 20 × 20 m^2^ randomly placed quadrats for tree species with DBH ≥ 10 cm in woody vegetation sites separated by at least 15 m between quadrat. Meanwhile, in each large quadrat, 5 × 5 m^2^ for shrub and liana species with ≤ 10 cm were nested (Figure S2). Thus, the following vegetation traits were measured accordingly: (1) by placing five 1 × 1 m^2^ quadrats (four in the corner and one in the center) in each nested quadrat; herb and fern species richness was identified and counted; (2) woody species richness and abundance were determined from the larger and nested plots; (3) woody species density (abundance of individual trees, shrubs and lianas/0.8 ha) was also estimated from each site; and (4) average tree canopy cover was estimated using GLAMA (Gap Light Analysis Mobile Application software) from vertically upward looking photos (approximately 8 photos/site) either directly collected in the field or retrieved photographs with a digital camera (Nikon D5300) from sampling sites (Gonsamo et al., 2011; Tichý, 2016).

Landscape connectivity metrics (landscape scale covariates), including elevation, distance to the nearest road (both paved and unpaved roads and trail with at least 1 m wide), distance to nearest water points, and distance to nearest settlements were gauged either directly at the site using a handheld GPS and tape meter or indirectly using Google earth images. Nearest and accessible metrics to some sites were measured in the field. Average on‐site ambient temperature and precipitation measurements would have been costly and very difficult to conduct in each site; instead, we obtained climatic data from NASA 2022 (https://power.larc.nasa.gov/data‐access‐viewer/) to understand species‐habitat associations.

### 2.5 Data analysis

Single‐season occupancy model was applied to understand the influence that habitat covariates have on occupancy and detectability while accounting for imperfect detection (MacKenzie et al., 2002, 2018). The detection history was derived from a sequence of species detection/non‐detection dichotomous data (i.e., detection = 1 and non‐detection = 0) that were pooled into occasions from consecutive camera days for each site. For occupancy models, data collected by camera traps needs to be divided into sampling occasions (Sollmann, 2018). Such data treatment is important to maximize detectability, maintain spatiotemporal independence among occasions and thereby increases adequacy of model fit. Sensitive analysis was conducted without incorporating any covariates to evaluate the discrepancy of occupancy and detection estimates for different sampling intervals. Based on the input of the analysis, we chose the balance between high parameter estimates and small confidence intervals (see Table S1). Consequently, an occasion was defined as an interval of two camera days for both study areas.

Cameras were active for approximately six consecutive days (*n* = 98, 2–10 days) to obtain an average of three occasions per site at GCCA area. Whereas cameras at SEA area were active for approximately eight consecutive days (*n* = 48, 4–12 days) to obtain an average of four occasions per site. Number of camera days varied depending on the probability of detection of the species in the two different areas. Such study duration is recommended for high detectable species (Guillera‐Arroita et al., 2010; MacKenzie & Royle, 2005). To account occupancy model assumptions (MacKenzie et al., 2002, 2018), each site was surveyed between one to five repeated occasions (*Κ*_max_ = 5; *Κ*_average_ = 2.95) in GCCA from March to June 2020, while in SEA each site was surveyed two to six repeated occasions (*Κ*_max_ = 6; *Κ*_average_ = 3.46) from February to March 2020. The discrepancy in number of occasions per site was due to accessibility, logistical constraints, security, weather conditions, and technical problems. We had missed observations in some sites meaning that sampling was not conducted at site *i* during time *t* and hence a missed observation represented by hyphen (−) was filled instead in the complete detection history (**h**_*i*_). This also included data from malfunctioned cameras and blank photos in some cameras.

We used PRESENCE program v.2.13.39 (Hines, 2006) to model occupancy and detection estimates. The parameters were estimated using logit link and a maximum likelihood approach in the program (MacKenzie et al., 2002, 2018). Occupancy probability (Ψ) was modeled as a logit link function of fine‐scale level and landscape scale covariates. The structure of model framework of the occupancy probability of a site (i) in association with the site‐specific covariates is expressed as:

(1) $$\text{logit}\;\left(\Psi_{i}\right)={ꞵ}_{0}+{ꞵ}_{1}X_{\text{i1}}+{ꞵ}_{2}X_{\text{i2}}+\ldots +{ꞵ}_{u}X_{\mathrm{iu}}$$

Likewise, the detection probability (ρ) was modeled as a logit link function of observation‐specific covariates. The logit equation derived from the probability of detecting a species at site *i*, during survey *j* in association with the covariates is:

(2) $$\text{logit}\;\left(\rho_{\mathrm{ij}}\right)={ꞵ}_{0}+{ꞵ}_{1}X_{\text{i1}}+{ꞵ}_{2}X_{\text{i2}}+\ldots +{ꞵ}_{u}X_{\mathrm{iu}}+{ꞵ}_{u+1}y_{\text{ij1}}\ldots {ꞵ}_{u+v}y_{\mathrm{ijv}},$$

where *X*_*i1*_ *… X*_*iu*_ refers to site covariates associated with the probability of a site *i* being occupied and *y*_*ij1*_ *… y*_*ijv*_ refers to sample covariates.

All continuous covariates were normalized by z score conversion (mean = 0 and SD = 1) to help convergence of the maximum likelihood algorithm prior to analysis (Schielzeth, 2010). Such data transformation produces better model performance and interpretability (Gelman & Hill, 2007; Schielzeth, 2010). Since we had spatial data, collinearity was assessed using variance inflation factor (VIF). Covariates with highest VIF were dropped in the analysis and covariates at threshold level VIF < 5 and Spearman's correlation (*r*_*s*_ < 0.7) were retained (Dormann et al., 2013; Zuur et al., 2010). Of the strongly correlated covariates, we retained ecologically important covariates based on field evidence and existed literature to understand their influence on occupancy and detectability. With a total of 11 covariates, the global model was run, and subsequently competing models were constructed based on plausible additive covariates. The null model (ψ(.),ρ(.)) was also constructed to compare with the relative weight of other additive models which included one or more covariates.

Since the ratio of effective sample size to the number of parameters (n/k) was small, model selection procedures were carried out using Akaike's Information Criterion for small sample bias adjustment (AIC_*c*_) from the competing candidate set of models (Burnham & Anderson, 2002), where the most supported models are top‐ranked models with ΔAIC_*c*_ ≤ 2.0 (Burnham & Anderson, 2002). Summed model weights of each covariate from each model were also calculated to rank the relative importance of the covariates (Burnham & Anderson, 2002). Then, in order to retain ecologically meaningful covariates, models with ΔAIC_*c*_ ≤ 4.0 were selected to drive model average estimates of occupancy and detection probabilities (Burnham et al., 2011) (Tables 2 and 3). Competitive models were used to estimate Ψ and ρ and calculated parameter estimates, standard errors (SEs), and level of significance based on 95% CI (zero‐overlapped method) for each covariate. Uninformative parameters (Arnold, 2010; Leroux, 2019) were also assessed in our model sets. Estimates of the slopes (i.e., β coefficients) for covariates were used to determine the magnitude of their influence on Ψ and ρ.

**TABLE 2:** Results of model selection for Moorland Francolins occupancy and detection probabilities in the central highlands of Ethiopia.

Model structure	AIC_*c*_	ΔAIC_*c*_	ω_*i*_	*Κ*	−2 L	ĉ
Traditionally protected landscape (GCCA)						
Ψ(Hsp + T_caco_ + Pre + DR),p(E + T + P)	257.40	0.00	0.08	9	237.35	0.88
Ψ(Hsp + Pre + DR),p(E + T + P)	257.46	0.06	0.07	8	239.84	0.83
Ψ(Hsp + Pre + Elev + DR),p(E + T + P)	258.01	0.61	0.06	9	237.96	0.88
Ψ(WD + Hsp + Pre + DR),p(E + T + P)	258.07	0.67	0.05	9	238.02	0.80
Ψ(Hsp + T_caco_ + Pre + DR),p(E + P)	258.16	0.76	0.05	8	240.54	0.81
Ψ(Hsp + Pre + Elev + DR),p(E + P)	258.39	0.99	0.05	8	240.77	0.83
Ψ(Hsp + Pre + DR),p(E + P)	258.44	1.04	0.05	7	243.20	0.84
Ψ(Hsp + T_caco_ + Pre + Elev + DR),p(E + T + P)	258.47	1.07	0.04	10	235.94	0.82
Ψ(Hsp + Pre + Elev),p(E + T + P)	258.94	1.54	0.04	8	241.32	0.88
Ψ(WD + Hsp + Pre + DR),p(E + P)	258.96	1.56	0.03	8	241.34	0.83
Ψ(WD + Hsp + Pre + Elev + DR),p(E + T + P)	259.17	1.77	0.03	10	236.64	0.83
Ψ(WD + Hsp + T_caco_ + Pre + DR),p(E + T + P)	259.18	1.78	0.03	10	236.65	0.80
Ψ(Hsp + T_caco_ + Pre + DR + DW),p(E + T + P)	259.35	1.95	0.03	10	236.82	0.77
…						
Ψ(.),ρ(.)	298.28	40.88	0.00	2	294.15	1.09
Human‐modified landscape (SEA)						
Ψ(Hsp + Tcaco + DR + DS),p(.)	182.77	0.00	0.07	6	168.72	0.99
Ψ(Hsp + DS),p(.)	183.32	0.55	0.06	4	174.39	1.14
Ψ(Tcaco + DS),p(.)	183.37	0.60	0.05	4	174.44	1.15
Ψ(Hsp + DR + DS),p(.)	183.62	0.85	0.05	5	172.19	0.97
Ψ(Hsp + Tcaco + DS),p(.)	183.75	0.98	0.04	5	172.32	1.15
Ψ(Tcaco + DS),p(E)	184.13	1.36	0.04	5	172.70	1.41
Ψ(Hsp + DS),p(T)	184.32	1.55	0.03	5	172.89	1.10
Ψ(Tcaco + DR + DS),p(.)	184.40	1.63	0.03	5	172.97	1.03
Ψ(Hsp + Tcaco + DR + DS),p(T)	184.45	1.68	0.03	7	167.65	0.95
Ψ(Hsp + DR + DS),p(T)	184.65	1.88	0.03	6	170.60	0.92
Ψ(Hsp + Tcaco + DR + DS),p(T)	184.68	1.91	0.03	7	167.88	1.04
Ψ(Tcaco),p(.)	184.76	1.99	0.03	3	178.21	1.15
…						
Ψ(.),ρ(.)	186.58	3.81	0.009	2	182.31	1.09

*Note*: Model rankings are based on the AIC_*c*_ values; AIC_*c*_ values compared to the top‐ranked model (ΔAIC_*c*_); ΔAIC_*c*_ scores ≤ 2.0 are the top‐ranked model; model weight (ω_*i*_), and number of parameters (*Κ*), and −2 L = −2Log_e_L. ĉ = overdispersion parameter to estimate lack of fit.

**TABLE 3:** Summed model weight (Σω_*i*_) and influence of covariates calculated from model‐averaged beta coefficient estimates and standard errors (β_mean_ ± SE).

Site	Covariate	Σω_*i*_	β_mean_ ± SE	95% CIs		*p*‐Value
				Lower	Upper	
GCCA	Occupancy (Ψ)					
	Predator	**0.97**	**−2.12 ± 0.84**	**−3.76**	**−0.48**	**.011**
	Herb species richness	**0.97**	**1.40 ± 0.68**	**0.07**	**2.74**	**.039**
	Distance to road	**0.78**	**−0.74 ± 0.35**	**−1.44**	**−0.05**	**.034**
	Tree canopy cover	0.46	−0.58 ± 0.37	−1.30	0.13	.117
	Elevation	0.35	0.79 ± 0.60	−0.39	1.97	.189
	Woody density	0.22	−0.46 ± 0.42	−1.29	0.37	.277
	Distance to water	0.10	0.21 ± 0.41	−0.59	1.00	.621
	Distance to settlement	0.06	0.36 ± 0.49	−0.60	1.33	.472
	Detection (ρ)					
	Occasion	**0.99**	**0.68 ± 0.23**	**0.23**	**1.13**	**.003**
	Precipitation	**0.92**	**0.75 ± 0.36**	**0.05**	**1.45**	**.037**
	Temperature	0.70	0.40 ± 0.23	−0.04	0.84	.082
SEA	Occupancy (Ψ)					
	Distance to settlement	**0.76**	**0.74 ± 0.41**	**−0.07**	**1.55**	**.071**
	Tree canopy cover	0.72	−0.84 ± 0.48	−1.77	0.09	.080
	Herb species richness	0.60	0.83 ± 0.48	−0.11	1.77	.083
	Distance to road	0.37	0.62 ± 0.41	−0.18	1.42	.131
	Predator	0.09	1.10 ± 1.23	−1.31	3.51	.378
	Woody density	0.03	0.23 ± 0.44	−0.63	1.09	.614
	Detection (ρ)					
	Occasion	0.26	0.39 ± 0.30	−0.19	0.98	.195
	Temperature	0.23	0.34 ± 0.27	−0.19	0.86	.210
	Precipitation	0.18	0.21 ± 0.21	−0.21	0.63	.322

*Note*: Lower and upper 95% confidence intervals of the coefficients were constructed. Non‐overlapping with zero (bold) shows significance values of β estimates.

We used a parametric bootstrap goodness of fit (GOF) using 10,000 permutations to assess the adequacy of fit of the global model (i.e., the most parameterized model) and Pearson's Chi‐square test (*χ* ^*2*^) and non‐Bayesian *p*‐value were implemented to check overdispersion (ĉ) (MacKenzie & Bailey, 2004). The degree of overdispersion parameter estimate (ĉ) or variance inflation factor was assessed using chi‐squared (GOF) statistic. It was calculated by dividing the observed test statistic by the average of simulated test statistics.

We computed the number of occasions (K) to enhance the odds of detecting Moorland Francolins in a site. We considered a set of four levels of confidence (ρ*): 0.7, 0.8, 0.9, and 0.99 by assuming that the species detection probability is always less than one. The occasion (K) was calculated from the detection probability (ρ) of the model averaging to determine the true absence of the species from a site (McGrath et al., 2015; Pellet & Schmidt, 2005; Sewell et al., 2010).

(3) $$K=\frac{\log\left(1-\rho *\right)}{\log\left(1-\rho \right)},$$

where ρ is the calculated detection probability and ρ* is the target detection probability as mentioned above.

Both original and square‐root transformed data were used sequentially for normality assumption using Shapiro–Wilk and homoscedasticity tests. Consequently, we used one‐way ANOVA to compare mean differences in photos captured among sampling months in GCCA, and a post hoc testing procedure using Bonferroni error adjustment was applied for multiple comparisons. We also used Mann–Whitney *U*‐test to compare mean differences in photos and parameter estimates across spatiotemporal. Similarly, this test was used for occupancy probability estimates comparison in relation to predator presence and absence. This data was analyzed in IBM SPSS statistics (version 20). A two‐tailed hypothesis test with an alpha value of 5% was considered.

## 3 RESULTS

### 3.1 Camera trapping in GCCA and SEA

**FIGURE 4:**
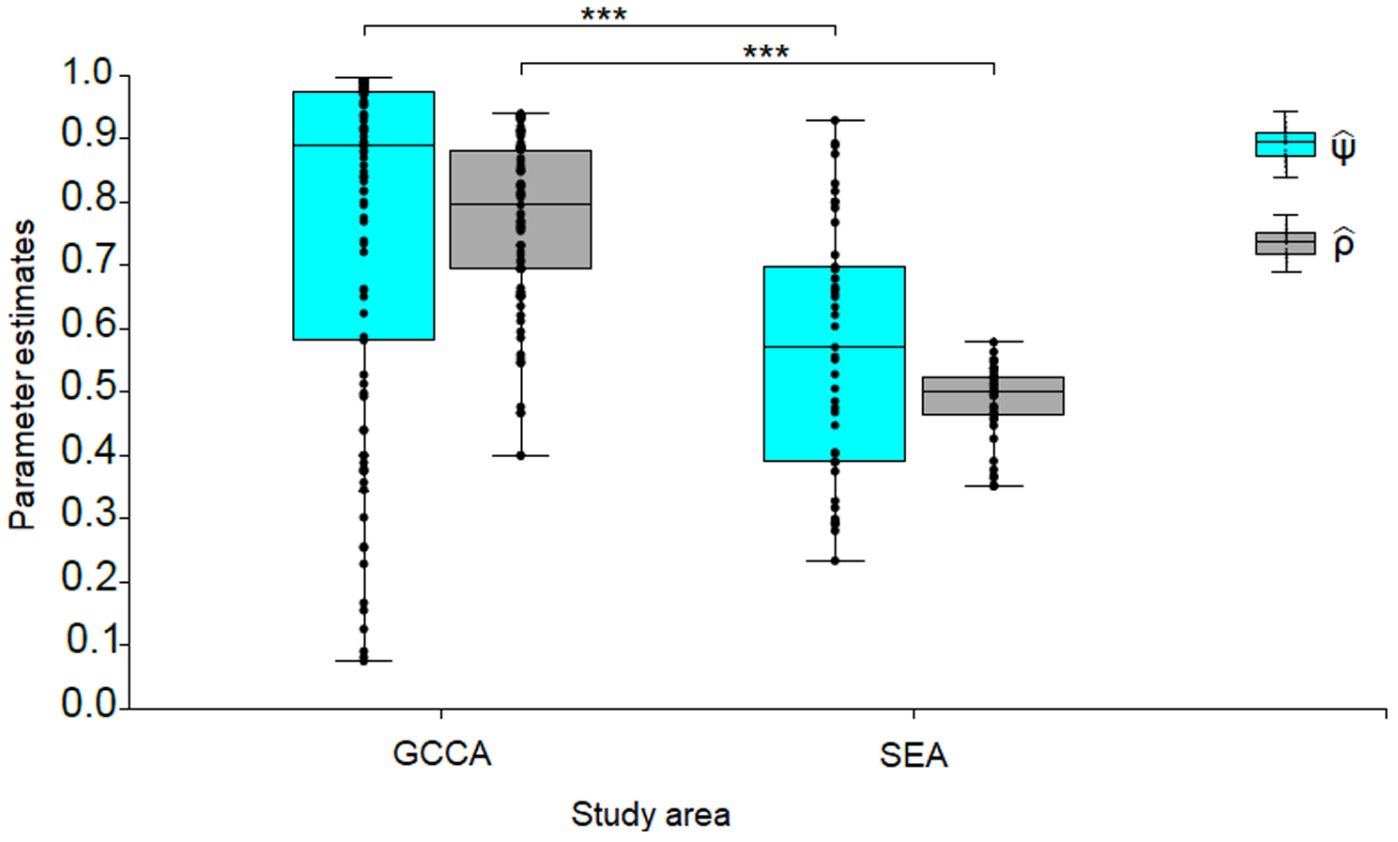
Parameter estimates (occupancy and detectability) of Moorland Francolin derived from model averaging. The asterisks (***) denote a strong statistically significant difference between parameter estimates in the study area at *p* < .001 level.

The deployed camera traps yielded 610 and 361 trap nights in GCCA and SEA, respectively. We failed to collect data from 21 (GCCA) and SEA (18) sites mostly due to battery failure and system malfunctioning. We found a significant difference in average photos captured among sampling months in GCCA (*F*_2,95_ = 11.775, *p* < .001). There was no significant difference in average photos captured between sampling months in SEA (Mann–Whitney test *U* = 277.5, *n* = 48, *p* = .893). Pooling the data across both study areas, the average photos captured in GCCA was approximately four units higher in comparison to SEA (Mann–Whitney test *U* = 1365, *n* = 146, *p* < .001) (Figure S3). Likewise, model‐averaged estimates of occupancy probability ($\hat{\psi }$) and detection probability ($\hat{\rho })$ parameters were significantly higher in the pristine habitat than in the disturbed landscape (Figure 4).

### 3.2 Habitat use modeling for traditionally managed habitat

We captured a total of 2632 photos (7–141 photos per site) from all sampling occasions in GCCA. Moorland Francolins were detected at 68 of 98 sites, which resulted in a naïve occupancy (proportion of sites that recorded at least one photograph on the whole camera sites) estimate of 0.69. In GCCA, at the habitat‐specific level, the findings showed that the highest habitat use was obtained in Mima Mound, *Euryops‐Alchemilla* shrubland, and *Helichrysum‐Festuca* grassland. Conversely, the least was shown across the tree belt (i.e., montane forest and *Eucalyptus* plantation) (Figure S4).

The null model (ψ(.), ρ(.)) appeared to be the least important model to explain the stochastic processes (Table 2; Table S2). The Ψ for this model was 0.73 (SE = 0.05) with a 95% CI of 0.63–0.82 and ρ of 0.85 (SE = 0.03) with 95% CI of 0.79–0.89. In GCCA, some evidence of breeding activity was observed from the camera traps, such as three juveniles were provisioned by both parents.

We constructed candidate sets without interactions between covariates to model Ψ and *р* in the order of parsimony models using ΔAIC_*c*_. The bootstrapping procedure and χ^*2*^ test revealed that the global model (ψ(WD + Hsp + T_caco_ + Pre + Elev + DR + DS + DW), ρ(E + T + P)) lacks overdispersion (χ^2^ = 35.95; *p* = .35; ĉ = 0.85), showing independence among sites. Subsequently, the combinations of occupancy and detection covariates of the top models were tested based on the lowest ΔAIC_*c*_ values. The bootstrapped top 13 models also showed adequate model fit (ĉ ~ 1, Table 2). The summed weight of the top‐ranked models (ΔAIC_*c*_ ≤ 2.0) was 0.61 and the most parsimonious model (ψ(Hsp + T_caco_ + Pre + DR),ρ(E + T + P)) had only 0.08 model weight, suggesting more plausible competing models existed to explain the occupancy and detection estimates (Table 2). We used model averaging to improve inference as the top model clearly showed model selection uncertainty (Symonds & Moussalli, 2011). Due to the ecological importance of individual covariates included in the top models, we discounted models with less than five ΔAIC_*c*_ to increase model weight (Richards, 2005) and we considered the top‐ranked models with summed model weight of 0.95 (Symonds & Moussalli, 2011).

**FIGURE 5:**
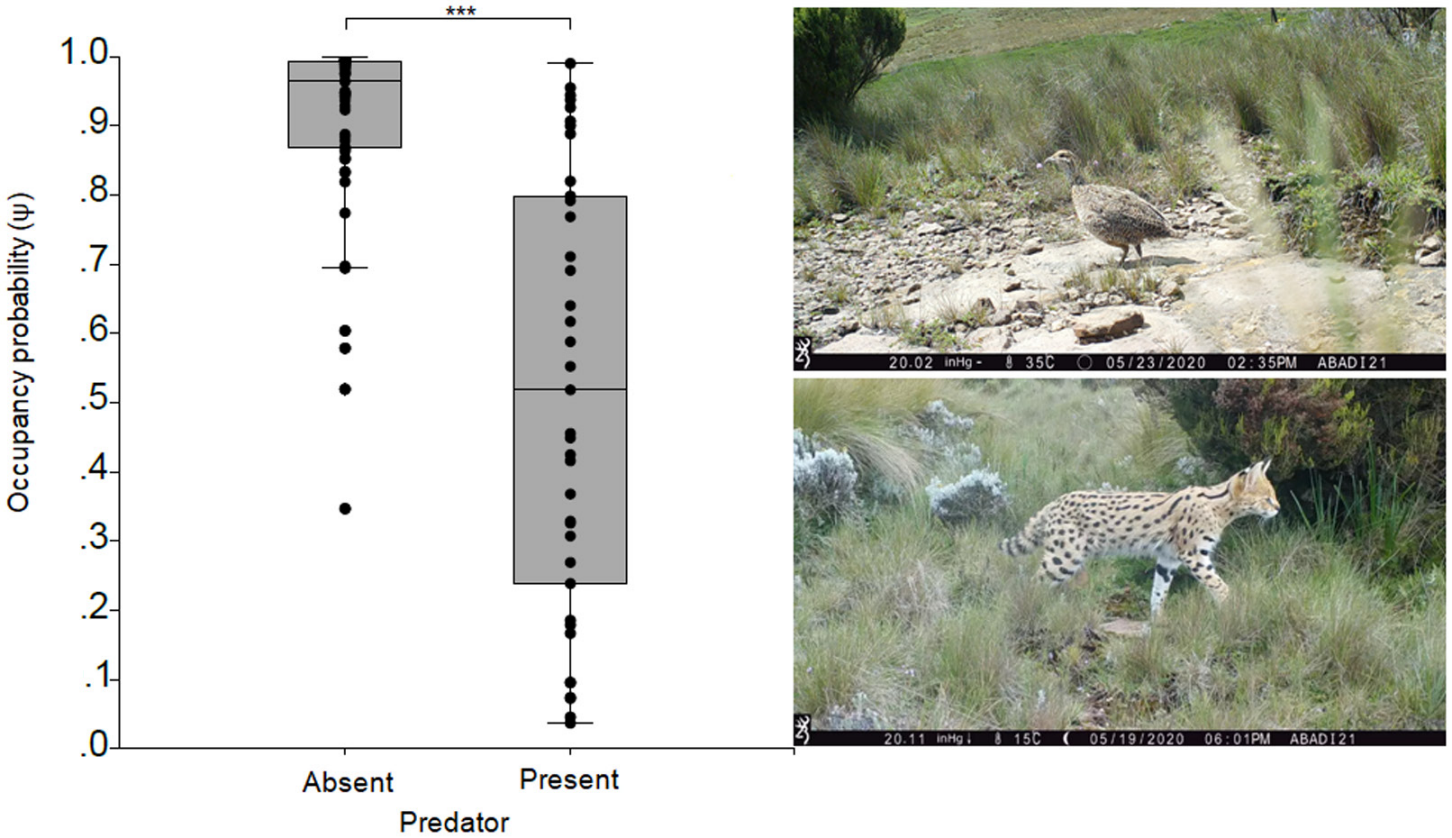
Occupancy probability of Moorland Francolin in association with predator presence/absence in GCCA. Cameras placed in woody plant species frequently had photos of predators like Serval *Leptailurus serval*. Error bars indicate standard errors of occupancy probability, ***p < .001.

Model‐averaged estimate of $\hat{\psi }$ across all sites was 0.76 (SD = 0.28) and $\hat{\rho }$ was 0.82 (SD = 0.05). The overall occupancy was 10% greater than the naïve occupancy estimates when detection probability is accounted for. As we hypothesized, predators negatively associated with the Ψ of Moorland Francolins in GCCA (β_mean_ ± SE = −2.12 ± 0.84; 95% CI: −3.76; −0.48) and the summed ω_*i*_ was 97% (Table 3). There was a higher average occupancy probability in the absence of predators in comparison to the presence of predators (Mann–Whitney *U*‐test = 244.5, *n* = 98, *p* < .001) (Figure 5). These predators were avian and mammalian species. We observed Yellow‐billed Kite *Milvus aegyptius*, Augur Buzzard *Buteo augur*, Verreaux's Eagle *Aquila verreauxii*, and Common Kestrel *Falco tinnunculus* to be common potential aerial predators of Moorland Francolins in the area. The most important potential mammalian predators were African Civet *Civettictis civetta*, Honey Badger *Mellivora capensis*, Black‐backed Jackal *Canis mesomelas*, Serval *Leptailurus serval* and White‐tailed Mongoose *Ichneumia albicauda*.

**FIGURE 6:**
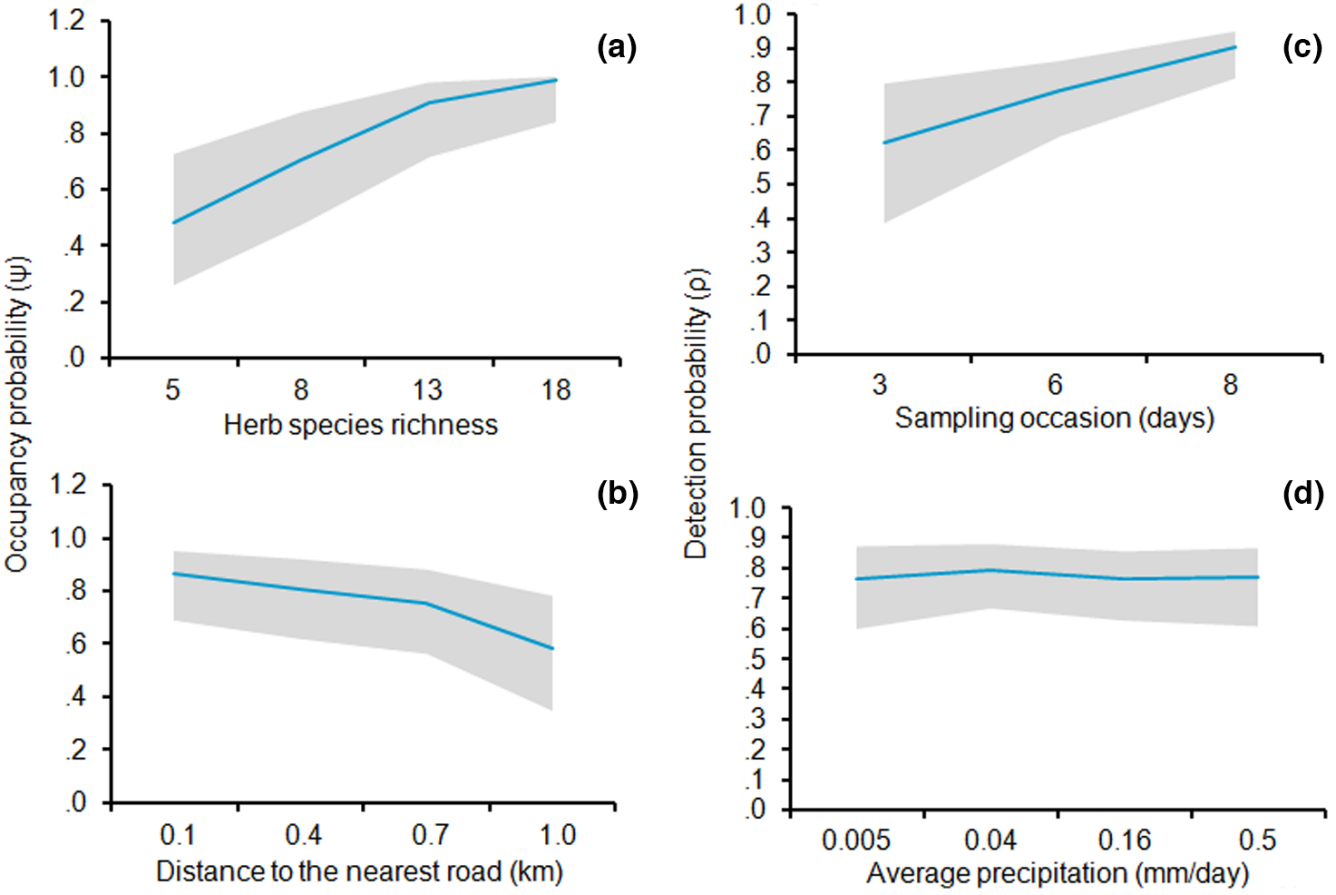
(a, b) Occupancy probability (ψ) of Moorland Francolin in association with herb species richness and distance to the nearest road (km) and (c, d) Detection probability (ρ) of the species in association with sampling occasion and average precipitation (mm/day), respectively. The estimates for the parameters are created from the most parsimonious model that holds these covariates and the shaded area in each graph shows 95% confidence intervals.

We also found that herb species richness showed a significantly positive influence the occupancy of the species based on model averaging estimates (β_mean_ ± SE = 1.40 ± 0.68, 95% CI: 0.07–2.74) and the summed ω_*i*_ was 97% (Table 3; Figure 6). Contrary to our prediction, distance to road was significantly negatively influenced the Ψ of the species and the model weight of the covariate was 78% (β_mean_ ± SE = −0.74 ± 0.35; 95% CI: −1.44, −0.05), suggesting that occupancy probability decreased as the distance to road increased in the pristine habitat (Table 3; Figure 6).

As depicted in the top models, the ability to detect Moorland Francolins was modeled as a function of survey occasion, precipitation, and temperature with summed model weight of 0.95, 0.92, and 0.70, respectively. The most important covariates supported by our hypotheses, however, included sampling occasion (β_mean_ ± SE = 0.68 ± 0.23, 95% CI: 0.23–1.13) and precipitation (β_mean_ ± SE = 0.75 ± 0.36, 95% CI: 0.05–1.45), both of which significantly positively influenced the detectability of the species (Table 3; Figure 6). Although the detectability of the species was increasing with temperature, the beta coefficient estimate (β_mean_ ± SE = 0.40 ± 0.23; 95% CI: −0.04 to 0.84) overlapped zero which exhibited a positive association but non‐significant difference with habitat use of the species.

### 3.3 Habitat use modeling for human‐modified landscape

In the human‐modified landscape, a total of 339 photos (2–29 photos per site) from 23 sites were trapped, yielding a naïve occupancy estimate of 0.48. The Ψ estimate without any covariate was 0.54 (SE = 0.08) with a 95% CI of 0.38–0.70 and ρ of 0.54 (SE = 0.06) with a 95% CI of 0.42–0.65. In this study area, based on the above considerations, the null model was included in the top important models with **ω**_***i***_ = 0.95 to explain the stochastic processes. The global model (ψ(WD + Hsp + T_caco_ + Pre + Elev + DR + DS + DW),ρ(E + T + P)) showed no evidence of lack of fit (χ^2^ = 118.13; *p* = .35; ĉ = 1.07). The most parsimonious model (Ψ(Sprich + T_caco_ + DR + DS),ρ(.)) had 0.07 model weight. Hence, all top models (ΔAIC_*c*_ ≤ 2.0) were equally supported to influence habitat use modeling in the case of SEA‐disturbed sites (Table 2; Table S2).

Model‐averaged estimate of $\hat{\psi }$ across all sites in SEA was 0.56 (SD = 0.19) and $\hat{\rho }$ was 0.48 (SD = 0.06). The overall occupancy was underestimated by approximately 17% when detection probability is not accounted for. Distance to settlement, tree canopy cover, herb species richness, distance to road, predator, and woody density appeared in the competing models to explain habitat use of the target species in this area. As predicted, distance to settlement (**ω**_***i***_ = 0.76; β_mean_ ± SE = 0.74 ± 0.41; 95% CI: −0.07 to 1.55) positively associated with habitat use of the species, yet its respective 95% CIs slightly overlapped zero. Other covariates also showed non‐significant associations with occupancy of the species (Table 3).

In this study area, detectability was more supported without covariates based on the top models. Thus, the sample covariates predicted to influence detectability had relatively low summed weight and 95% of CIs overlapped zero. In this disturbed habitat, detection probability was not significantly affected by sample covariates but all covariates depicted positive association with detectability. The summed model weight of each covariate was below 0.30 (Table 3).

### 3.4 Recommended number of sampling occasions (K)

The sampling occasion (K) needed at GCCA was ranged from 1 to 3, this meaning that a single occasion (mean 0.86 and 1.14, respectively) was needed for a targeted confidence level of probabilities of 0.7 and 0.8 and two (mean 1.64) and three (mean 3.27) occasions sequentially were sufficient for 0.9 and 0.99 detection probabilities to estimate the true absence of the species at a given site. Similarly, we found that 2, 3, 4, and 7 occasions sequentially were needed at SEA.

## 4 DISCUSSION

### 4.1 Occupancy and detection probability estimates using camera trap

Our study delivers the first insights into the habitat use of Moorland Francolins using a camera trap approach. Camera traps for this elusive and cryptic species helped us to avoid false‐positive detection, which also corroborates the respective assumption for the occupancy model. The overall or true occupancy estimates in both study areas were greater than the naïve occupancy (ψ) estimates when detection probability is accounted for. These suggest that models incorporate imperfect detections to discount underestimating of overall occupancy (Guillera‐Arroita et al., 2014; MacKenzie et al., 2018). Since we had small sample sizes and low density of individuals in SEA, we increased the sampling by one more occasion to minimize the effect of false‐negative detections of the target species. Increasing of sampling occasion helps to increase the precision and accuracy of detectability of species (MacKenzie & Royle, 2005; Moore et al., 2014).

In many tropical African countries, the protected areas are called “paper parks”‐existing in name only as they poorly counter habitat and species loss (Dudley & Stolton, 1999). However, GCCA as a traditionally protected area is exceptional in this case as the indigenous knowledge for conservation of natural resources, the Qero system, has supported several wildlife species for almost four centuries (Ashenafi & Leader‐Williams, 2005; Nigussie et al., 2019). Occupancy and detection probability estimates of Moorland Francolins were higher in traditionally protected areas than in unprotected areas, suggesting the persistent and high conservation effort supporting the Ethiopian Wolf (*Canis simensis*) by the local community in association with international organizations signifies the integrity and functionality of the whole community. Flagship species like this play a vital role in biodiversity conservation at local and global scales (Jarić et al., 2023), which is demonstrated by its positive side effects for Moorland Francolins and other species in GCCA, too. Unlike other carnivore species, this species is a rodent specialist (Ashenafi et al., 2012; Atickem & Stenseth, 2022; Vial et al., 2011).

### 4.2 Determinants of occupancy and detection probabilities

Based on beta estimates and moderate model weight, Moorland Francolins revealed an aversion to montane forest habitat due to the presence of predators in the tree canopies. The Afroalpine highlands are suitable habitats for predators (Clouet et al., 2000), and habitat use of many ground‐dwelling birds is negatively influenced by the presence of predators in and around the forest habitats (Abrha et al., 2018; Sukumal et al., 2017). In concordance with these findings, our results confirm that predators (both aerial and ground predators) may strongly negatively influence the habitat use of Moorland Francolins in GCCA, although the main diet of several raptors is rodents (Clouet et al., 2000).

Though hunting pressure is one of the key factors for decreasing francolin populations nationwide (Abrha et al., 2017; Gedeon, Rödder, et al., 2017; Töpfer et al., 2014) and globally (McGowan et al., 2012), this threat was only of minor importance to Moorland Francolins in GCCA. However, in both study areas, but essentially in SEA, hunters preferably target to capture Erckel's Francolin *Pternistis erckelii* that usually subsist in habitats below the tree line in GCCA (Demis & Tesfaye, pers. comm.), and sympatrically with Moorland Francolins in SEA. Hunting pressure apparently is much more pronounced on *P. erckelii* due to its larger size and because of the different perceptions by the local communities toward both highland francolins.

Herb species richness was also supported based on model weight and top models. The protected grassland of GCCA covers almost 60% of its total area (Steger et al., 2020) and holds several range‐restricted species (Ashenafi et al., 2012; Ashenafi & Leader‐Williams, 2005). As expected, the occupancy probability of Moorland Francolins increased with herb species richness in GCCA, in line with other reports on pheasant species (Jolli et al., 2012; Sukumal et al., 2017). This vegetation type is widespread in the plateau of Afroalpine biome of Ethiopia (Nigussie et al., 2019; Steger et al., 2020) and it is the source of food and provides essential shelter for many grassland specialists (Töpfer & Gedeon, 2020). It had also a positive influence on the habitat use of Moorland Francolins at SEA, but the 95% confidence interval of the β‐coefficient estimate overlapped zero showing less support for its influence on the species. This is because the area has been increasingly transformed into a monocultural plantation (Bahru et al., 2021; Tadesse & Tafere, 2017), and is subject to tourism activities (Asefa, 2018; Tesema & Berhan, 2019), overgrazing and other human‐induced disturbances in the plateau of central highlands (Asefa et al., 2020). For instance, a recent report showed that the natural grassland of Entoto Natural Park has decreased over the last three decades and that the area is now dominated by an *Eucalyptus* plantation (Tesema & Berhan, 2019). In such areas, Moorland Francolins showed a pronounced aversion toward modified habitat types. This implies that Afromontane grassland and shrubland specialists, especially Moorland Francolins might gradually become locally extinct.

Distance to road was also the other strongest covariate influencing the occupancy probability of Moorland Francolins, similar to other reports in ground‐dwelling bird species (Whitworth et al., 2018). The occupancy probability of the species was higher along the edge of roadsides and trails than at sites located in remote in GCCA, in concordance with other reports on wildlife species (Kroeger et al., 2022; Paemelaere et al., 2023). This is unexpected because roads can attract hunters and predators, delivering also other human‐induced perturbations (Dean et al., 2019; Kroeger et al., 2022). In GCCA, we observed that proximity to road attracts the species as there were food items mainly on the unpaved road, including grains and fruits thrown through window by passengers. Most roadsides have also dense native herbaceous vegetation, which may also help Moorland Francolins to survive. On the contrary, occupancy increased as the distance to road increased in SEA habitat but did not show a significant association with roads. This suggests that Moorland Francolins avoid roads and trails in a human‐modified landscape. Thus, roads may have positive effects on bird species in more pristine habitats (Kroeger et al., 2022) and in areas where hunting pressure is controlled as a management strategy (Whitworth et al., 2018). Local low temperatures and high ground vegetation cover (Nigussie et al., 2019; Steger et al., 2020) may lead the species to use the roadsides and trails: (1) to enhance foraging opportunities; (2) to stay more vigilant to avoid risk of predation; (3) as a heat source; (4) to facilitate mating, connectivity and communication.

Avoidance of human settlements is likely related to livestock grazing causing herb species richness to shrink at the GCCA periphery (i.e., human occupation). Similarly, the effect of distance to settlement as a type of human disturbance posed a positive effect on Moorland Francolins in SEA. There was no significant difference for the covariate in this area, yet relatively high model‐averaged beta coefficient estimate; model weight and confidence intervals reveal irregularity in association with the species, most presumably due to lack of habitat heterogeneity, a small sample size, limited number of cameras, and small sampling occasions, as compared to recommended occasions. Hence distance to settlement had a slightly significant positive influence on the species in SEA, agreeing with previous studies on pheasants (Chen et al., 2019; Jolli et al., 2012; Nuttall et al., 2017; O'Brien & Kinnaird, 2008), other bird (Pardo et al., 2017) and mammal species (Paemelaere et al., 2023; Semper‐Pascual et al., 2020).

In line with our hypothesis, sampling occasion significantly positively influences the detectability of the species in GCCA. Conversely, in SEA, this covariate appeared in one of the most parsimonious models and it positively influenced detectability but it had low model weight and the beta coefficient estimates showed statistically non‐significance association. The detectability may be affected by spatial variations and sample sizes. Our hypothesis that species detection increases with number of days of cameras deployed showed consistency with other findings in bird (Paemelaere et al., 2023; Si et al., 2014) and mammal species (Holzner et al., 2021; Semper‐Pascual et al., 2020; Shannon et al., 2014; Si et al., 2014; Wevers et al., 2021). The magnitude of sampling occasion on detection probability estimate demonstrates species‐specific response (Iannarilli et al., 2021).

In Ethiopia, after a long dry season, both a small and a main rain season occurs in most highland areas (Mohammed et al., 2022). Several francolin species are adapted to this seasonally changing precipitation regime (Abrha et al., 2018; Gedeon, Rödder, et al., 2017), which allows the areas to replenish food resources and ecosystem greenness vital for breeding (Abrha et al., 2018). This is because francolins may find plenty of food by easily raking and scratching the wet ground (Abrha et al., 2018). Moreover, during rain seasons, birds of prey soar less, and agro‐pastoral encroachments seem lower compared to the dry season (pers. obs). Elsewhere in tropics, the breeding season of birds is reported to be associated with the beginning of precipitation and this is linked to the abundance in food and cover resources (Cox et al., 2013; França et al., 2020; Jansen & Crowe, 2005). In our species, some camera traps have documented chicks being fed by their parents in GCCA, and this implies that the breeding season of the species may coincide with the short and mild precipitation distribution from February to June. Similarly, temperature positively influenced the detectability of the species, but there was little support for our hypothesis based on models. This may suggest that the species avoids extreme temperatures. Collectively, climate factors are very important for the detectability of the target species in the central highlands of Ethiopia.

### 4.3 Camera trapping for assessment of cryptic bird species

The Moorland Francolins, similar to other pheasants in the region, could potentially go visually undetected, particularly in areas of low population density and in disturbed habitats. Extreme weather conditions, seasonality, expert experience, and other factors may also obscure the ability of detecting the species. This is because the birds usually remain silent, hidden, and squatted when people approach them. Thus, false‐negative detection could bias inferences about the occupancy and detection probability estimates and other parameters. However, the deployment of non‐invasive modern approaches like remotely triggered camera traps can avoid such ecological concerns. This approach also helps to discover new geographical ranges, other wildlife species (including predators) and thereby helping to understand the interactions of the Moorland Francolins in its natural habitat. Another positive feature of the camera trapping technique is that it is cost and time‐effective. Our results strongly support the deployment of camera traps for the detection of cryptic and little‐known species in a topographically complex region. Camera traps provide reliable comprehension and precision of occupancy of Moorland Francolins in the Afroalpine Biome. Such camera trap data (O'Brien & Kinnaird, 2008; Sharief et al., 2022; Si et al., 2014; Steenweg et al., 2017; Wearn & Glover‐Kapfer, 2017) ultimately promotes the proper conservation of the target species.

## 5 CONCLUSIONS

The findings demonstrate that habitat use of Moorland Francolins is higher in the more pristine habitats compared to the strongly human‐influenced in SEA. This suggests that a community‐based conservation area (i.e., GCCA) is a crucial remnant habitat of endangered and data‐deficient wildlife species in Ethiopia. Since such community‐based conservation approaches obviously support sustainable species‐habitat conservation, strengthening the existing Qero system and expanding the model to other potential hotspot sites and/or IBAs is strongly recommended to circumvent the mounting anthropogenic disturbances in the region (Asefa et al., 2017; Chengere et al., 2022; Razgour et al., 2021; Rodrigues et al., 2021).

Our results also show that the species uses various herb species, roadsides and trails for resting, hiding, survival, and reproduction. Conversely, predators threatened the francolins predominantly in native and plantation forests, thus Moorland Francolins tend to avoid tree canopy cover and human settlements in both study areas. In the human‐modified SEA areas, most covariates had a weak influence on the occupancy and detection estimates of our target species because habitats are dominated by *Eucalyptus* plantations, fragmented meadow hill patches, and farmlands, unlike the heterogeneous and protected habitats in GCCA.

We confirm that camera trap deployment corroborates the presence or absence of shy ground‐dwelling birds not only in known areas but also in understudied areas. The detectability of francolins was determined by the sampling occasion and precipitation. Further research using single or multi‐season modeling is required to understand the influence of habitat covariates, seasonal colonization, and local extinction from spatiotemporally replicated surveys.

## AUTHOR CONTRIBUTIONS

**Abadi Mehari Abrha:** Conceptualization (equal); data curation (lead); formal analysis (lead); investigation (lead); methodology (equal); project administration (equal); software (lead); validation (equal); visualization (equal); writing – original draft (lead); writing – review and editing (equal). **Kai Gedeon:** Conceptualization (equal); funding acquisition (equal); methodology (supporting); project administration (supporting); supervision (equal); writing – original draft (supporting); writing – review and editing (equal). **Lars Podsiadlowski:** Methodology (supporting); project administration (supporting); supervision (equal); writing – review and editing (equal). **Demis Mamo Weldesilasie:** Data curation (supporting); project administration (supporting); writing – original draft (supporting); writing – review and editing (supporting). **Till Töpfer:** Conceptualization (equal); funding acquisition (equal); methodology (supporting); project administration (equal); supervision (equal); validation (equal); visualization (equal); writing – original draft (supporting); writing – review and editing (equal).

## CONFLICT OF INTEREST STATEMENT

The authors declare that they have no conflict of interest.

## Supporting information


Appendix S1.

## Data Availability

The data that support the findings of this study are available from the corresponding author upon reasonable request.
