# Supplementary material for: Occupancy of the Ethiopian endemic Moorland Francolin in pristine and degraded Afroalpine biome using a camera trap approach
Source: Ecol Evol. 2023 Oct 31;13(11):e10551. doi: 10.1002/ece3.10551 (PMC10617016; doi:10.1002/ece3.10551)
Supplement: Supplementary file 1 — Appendix S1. [file ECE3-13-e10551-s001.zip › ece310551-sup-0001-Supinfo.docx]

**Supplementary information**

**Occupancy of the Ethiopian endemic Moorland Francolin in pristine and degraded Afroalpine biome using a camera trap approach**

FIGURE S1 Climatic conditions of GCCA and SEA from 1981-2020 (<https://power.larc.nasa.gov/data-access-viewer>). Temperature (°C) and precipitation (mm) values are based on mean monthly data for each year (mean maximum, average and mean minimum temperatures).


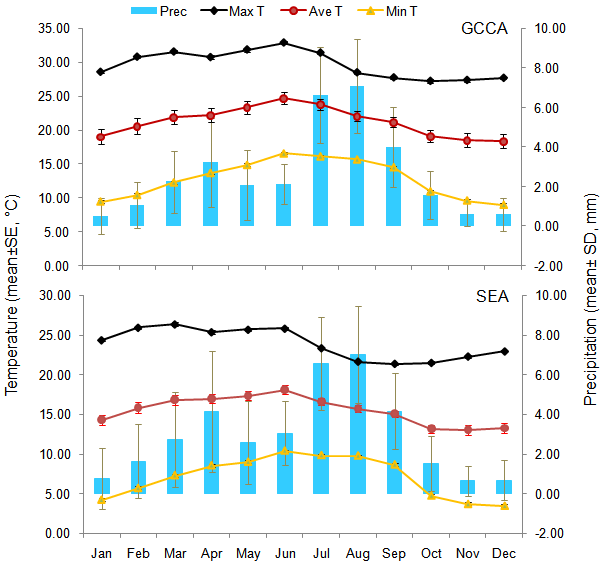


TABLE S1 Sensitive analyses for the constant model (null model) using different sampling occasions for GCCA and SEA study areas. The middle sampling occasion was opted over the lowest and highest estimates. Abbreviations: *k-*parameter; ψ-occupancy probability; ρ-detection probability, and n-effective sample size.

| Study area | | Sampling occasion | | | | | | | | | | | |
| --- | --- | --- | --- | --- | --- | --- | --- | --- | --- | --- | --- | --- | --- |
|  |  | Output 1 | | | | Output 2 | | | Output 3 | | | | |
| GCCA | *k* | Estimate | SE | 95% CI | n | Estimate | SE | 95% CI | n | Estimate | SE | 95% CI | n |
|  | ψ | 0.72 | 0.05 | 0.61-0.80 | 98 | 0.73 | 0.05 | 0.63-0.82 | 98 | 0.77 | 0.05 | 0.66-0.85 | 94 |
|  | ρ | 0.63 | 0.02 | 0.59-0.68 | 98 | 0.85 | 0.02 | 0.79-0.89 | 98 | 0.86 | 0.03 | 0.79-0.91 | 94 |
| SEA | ψ | 0.51 | 0.08 | 0.36-0.65 | 48 | 0.54 | 0.08 | 0.38-0.70 | 48 | 0.54 | 0.08 | 0.37-0.69 | 48 |
|  | ρ | 0.38 | 0.04 | 0.31-0.46 | 48 | 0.54 | 0.06 | 0.42-0.65 | 48 | 0.65 | 0.07 | 0.50-0.77 | 48 |

FIGURE S2 Schematic illustration of quadrant and camera placement in each circular plot (c. 0.8 ha). All quadrats for woody and herbaceous plant species (A) and camera trap mounted on coarser grass species (B) are shown.


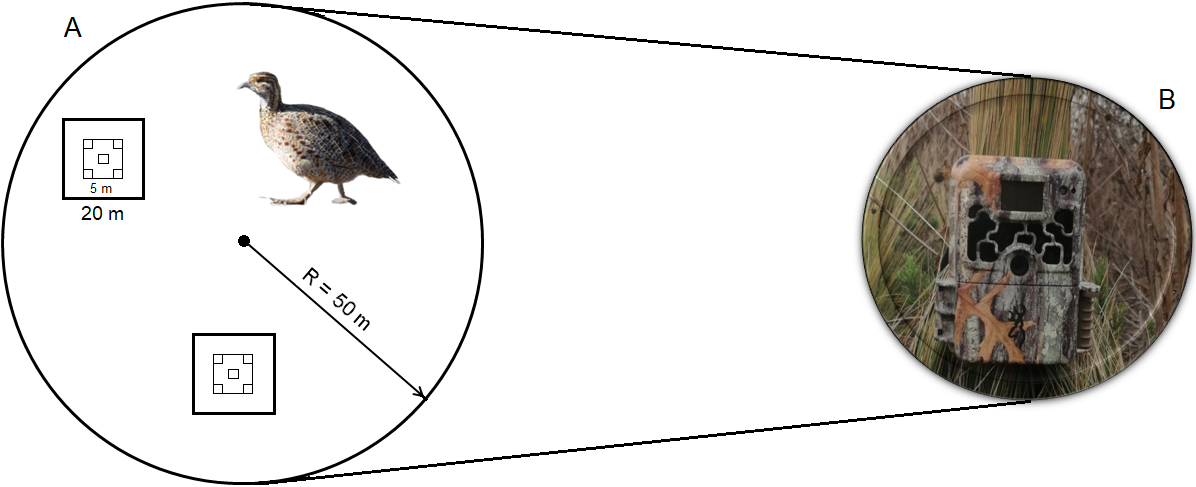


FIGURE S3 Number of photos captured in different sampling months and across study area. The asterisks (***) denote p < 0.001 level.


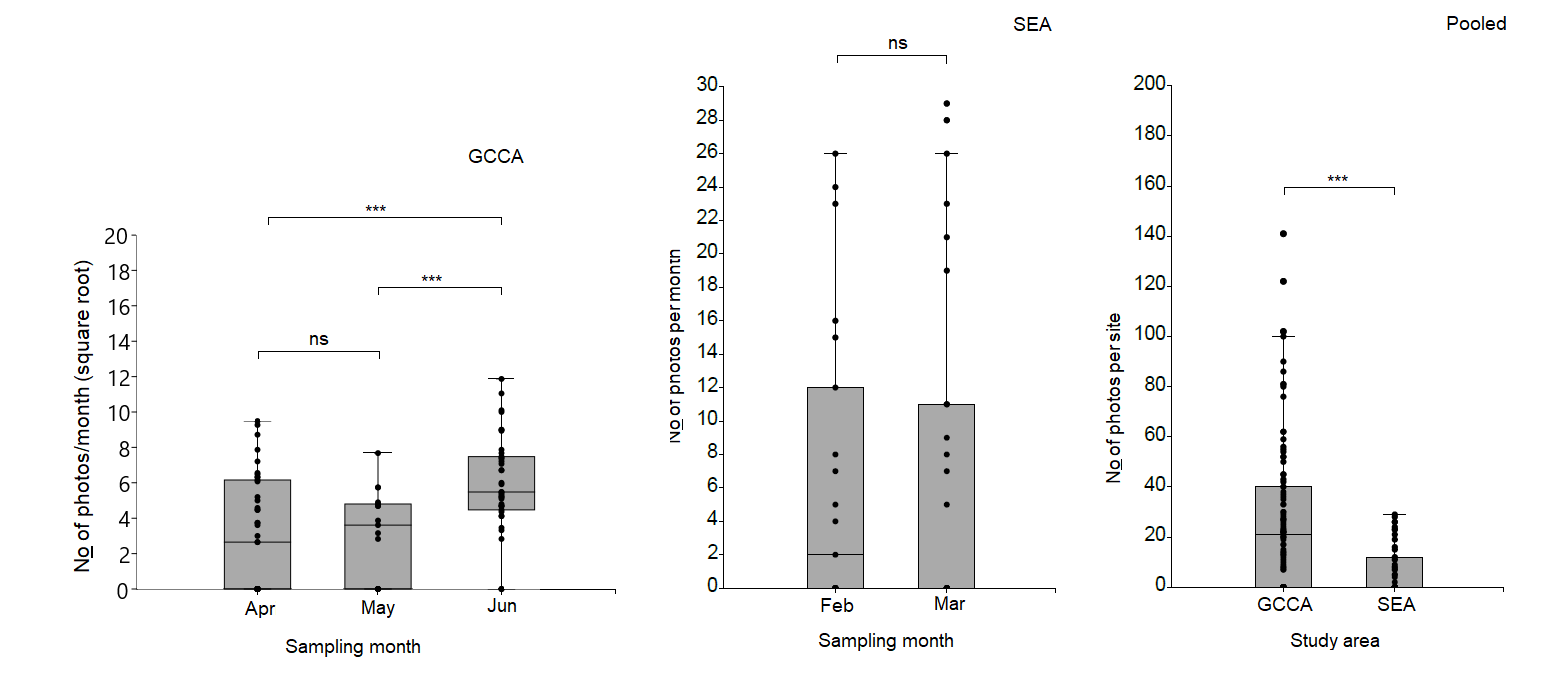


FIGURE S4 Occupancy and detection probability estimates of Moorland Francolin in different habitat types in central highlands of Ethiopia. Both parameter estimates were derived from model average estimates and error bars represent SEs. Abbreviations: FG-Festuca Grassland; HFG-Helichrysum-Festuca Grassland; EM-Erica Moorland; EAS-Euryops-Alchemilla Shrubland; Peat-Peatland; MM-Mima Mound and MF-Montane Forest


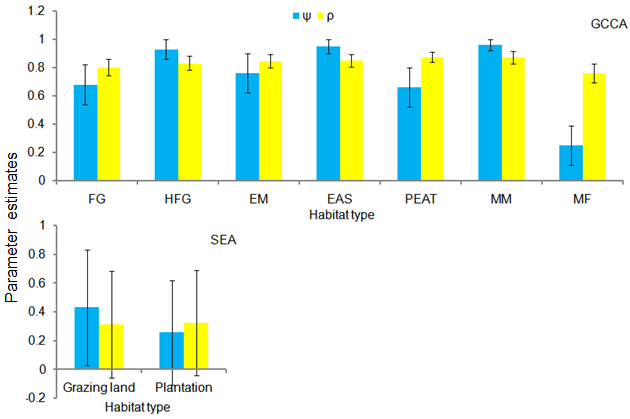


TABLE S2 Model outputs for GCCA and SEA study areas.

| **GCCA** | | | | | |
| --- | --- | --- | --- | --- | --- |
| **Model** | **AIC_c_** | **ΔAIC_c_** | **ω*_i_*** | ***Κ*** | **-2L** |
| psi(Hsp+Tcaco+Pre+DR),p(E+T+P) | 257.40 | 0.00 | 0.0801 | 9 | 237.35 |
| psi(Hsp+Pre+DR),p(E+T+P) | 257.46 | 0.06 | 0.0777 | 8 | 239.84 |
| psi(Hsp+Pre+Elev+DR),p(E+T+P) | 258.01 | 0.61 | 0.0590 | 9 | 237.96 |
| psi(WD+Hsp+Pre+DR),p(E+T+P) | 258.07 | 0.67 | 0.0573 | 9 | 238.02 |
| psi(Hsp+Tcaco+Pre+DR),p(E+P) | 258.16 | 0.76 | 0.0548 | 8 | 240.54 |
| psi(Hsp+Pre+Elev+DR),p(E+P) | 258.39 | 0.99 | 0.0488 | 8 | 240.77 |
| psi(Hsp+Pre+DR),p(E+P) | 258.44 | 1.04 | 0.0476 | 7 | 243.20 |
| psi(Hsp+Tcaco+Pre+Elev+DR),p(E+T+P) | 258.47 | 1.07 | 0.0469 | 10 | 235.94 |
| psi(Hsp+Pre+Elev),p(E+T+P) | 258.94 | 1.54 | 0.0371 | 8 | 241.32 |
| psi(WD+Hsp+Pre+DR),p(E+P) | 258.96 | 1.56 | 0.0367 | 8 | 241.34 |
| psi(WD+Hsp+Pre+Elev+DR),p(E+T+P) | 259.17 | 1.77 | 0.0331 | 10 | 236.64 |
| psi(WD+Hsp+Tcaco+Pre+DR),p(E+T+P) | 259.18 | 1.78 | 0.0329 | 10 | 236.65 |
| psi(Hsp+Tcaco+Pre+DR+DW),p(E+T+P) | 259.35 | 1.95 | 0.0302 | 10 | 236.82 |
| psi(Tcaco+Pre+Elev+DR),p(E) | 259.45 | 2.05 | 0.0287 | 7 | 244.21 |
| psi(Hsp+Pre+Elev),p(E+P) | 259.48 | 2.08 | 0.0283 | 7 | 244.24 |
| psi(Hsp+Tcaco+Pre+Elev),p(E+T+P) | 259.81 | 2.41 | 0.0240 | 9 | 239.76 |
| psi(Hsp+Pre+DR),p(E) | 259.84 | 2.44 | 0.0237 | 6 | 246.92 |
| psi(Hsp+Tcaco+Pre),p(E+T+P) | 259.87 | 2.47 | 0.0233 | 8 | 242.25 |
| psi(Hsp+Pre+DR+DW),p(E+T+P) | 259.89 | 2.49 | 0.0231 | 9 | 239.84 |
| psi(Hsp+Pre),p(E+T+P) | 260.12 | 2.72 | 0.0206 | 7 | 244.88 |
| psi(Hsp+Tcaco+Pre+DR),p(E) | 260.20 | 2.80 | 0.0198 | 7 | 244.96 |
| psi(WD+Hsp+Tcaco+Pre+Elev+DR),p(E+T+P) | 260.46 | 3.06 | 0.0173 | 11 | 235.39 |
| psi(Hsp+Tcaco+DR),p(E+T+P) | 260.52 | 3.12 | 0.0168 | 8 | 242.90 |
| psi(WD+Hsp+Pre+DR+DW),p(E+T+P) | 260.54 | 3.14 | 0.0167 | 10 | 238.01 |
| psi(Hsp+Tcaco+Pre+Elev+DR+DW),p(E+T+P) | 260.76 | 3.36 | 0.0149 | 11 | 235.69 |
| psi(WD+Hsp+Pre+Elev),p(E+T+P) | 260.88 | 3.48 | 0.0141 | 9 | 240.83 |
| psi(Hsp+Tcaco+Pre),p(E+P) | 260.92 | 3.52 | 0.0138 | 7 | 245.68 |
| psi(Hsp+Tcaco+Pre+Elev+DR+DS),p(E+T+P) | 261.00 | 3.60 | 0.0132 | 11 | 235.93 |
| psi(Hsp+Pre+DS),p(E+T+P) | 261.02 | 3.62 | 0.0131 | 8 | 243.40 |
| psi(Hsp+Tcaco+Pre+DR+DW+DS),p(E+T+P) | 261.20 | 3.80 | 0.0120 | 11 | 236.13 |
| psi(Hsp+Tcaco+Pre+DW),p(E+T+P) | 261.24 | 3.84 | 0.0117 | 9 | 241.19 |
| psi(Hsp+Pre),p(E+P) | 261.29 | 3.89 | 0.0115 | 6 | 248.37 |
| psi(Hsp+Tcaco+DR+DS),p(E+T+P) | 261.35 | 3.95 | 0.0111 | 9 | 241.30 |
| psi(.),p(.) | 298.28 | 40.88 | 0.0000 | 2 | 294.15 |
| **SEA** | | | | | |
| **Model** | **AIC_c_** | **ΔAIC_c_** | **ω*_i_*** | ***Κ*** | **-2L** |
| psi(Hsp+Tcaco+DR+DS),p(.) | 182.77 | 0.00 | 0.07 | 1.00 | 6 |
| psi(Hsp+DS),p(.) | 183.32 | 0.55 | 0.06 | 0.76 | 4 |
| psi(Tcaco+DS),p(.) | 183.37 | 0.60 | 0.05 | 0.74 | 4 |
| psi(Hsp+DR+DS),p(.) | 183.62 | 0.85 | 0.05 | 0.65 | 5 |
| psi(Hsp+Tcaco+DS),p(.) | 183.75 | 0.98 | 0.04 | 0.61 | 5 |
| psi(Tcaco+DS),p(E) | 184.13 | 1.36 | 0.04 | 0.51 | 5 |
| psi(Hsp+DS),p(T) | 184.32 | 1.55 | 0.03 | 0.46 | 5 |
| psi(Tcaco+DR+DS),p(.) | 184.40 | 1.63 | 0.03 | 0.44 | 5 |
| psi(Hsp+Tcaco+DR+DS),p(T) | 184.45 | 1.68 | 0.03 | 0.43 | 7 |
| psi(Hsp+DR+DS),p(T) | 184.65 | 1.88 | 0.03 | 0.39 | 6 |
| psi(Hsp+Tcaco+DR+DS),p(P) | 184.68 | 1.91 | 0.03 | 0.38 | 7 |
| psi(Tcaco),p(.) | 184.76 | 1.99 | 0.03 | 0.37 | 3 |
| psi(Tcaco),p(E) | 184.94 | 2.17 | 0.02 | 0.34 | 4 |
| psi(Tcaco+DS),p(P) | 184.98 | 2.21 | 0.02 | 0.33 | 5 |
| psi(Pre+Tcaco+DS),p(.) | 185.11 | 2.34 | 0.02 | 0.31 | 5 |
| psi(Hsp),p(.) | 185.12 | 2.35 | 0.02 | 0.31 | 3 |
| psi(Hsp+DR+DS),p(P) | 185.22 | 2.45 | 0.02 | 0.29 | 6 |
| psi(Hsp+Tcaco+DS),p(P) | 185.30 | 2.53 | 0.02 | 0.28 | 6 |
| psi(Hsp+Tcaco+DR+DS),p(E) | 185.33 | 2.56 | 0.02 | 0.28 | 7 |
| psi(Hsp+Tcaco),p(.) | 185.37 | 2.60 | 0.02 | 0.27 | 4 |
| psi(Hsp+Tcaco+DS),p(E) | 185.39 | 2.62 | 0.02 | 0.27 | 6 |
| psi(Hsp+Tcaco+DS),p(T) | 185.40 | 2.63 | 0.02 | 0.27 | 6 |
| psi(Hsp+DS),p(E) | 185.41 | 2.64 | 0.02 | 0.27 | 5 |
| psi(Tcaco+DS),p(E+T) | 185.43 | 2.66 | 0.02 | 0.26 | 6 |
| psi(Tcaco),p(E+T) | 185.44 | 2.67 | 0.02 | 0.26 | 5 |
| psi(Hsp+DR),p(.) | 185.52 | 2.75 | 0.02 | 0.25 | 4 |
| psi(Tcaco+Pre+DR+DS),p(.) | 185.58 | 2.81 | 0.02 | 0.25 | 6 |
| psi(WD+Tcaco+DS),p(.) | 185.63 | 2.86 | 0.02 | 0.24 | 5 |
| psi(Hsp+DS),p(E+T) | 185.65 | 2.88 | 0.02 | 0.24 | 6 |
| psi(Tcaco+DS),p(E+P) | 185.68 | 2.91 | 0.02 | 0.23 | 6 |
| psi(Pre+Tcaco),p(.) | 185.82 | 3.05 | 0.02 | 0.22 | 4 |
| psi(Tcaco+DR+DS),p(E) | 185.86 | 3.09 | 0.02 | 0.21 | 6 |
| psi(Hsp+Pre+Tcaco+DS),p(.) | 186.00 | 3.23 | 0.01 | 0.20 | 6 |
| psi(Hsp+DR+DS),p(E | 186.19 | 3.42 | 0.01 | 0.18 | 6 |
| psi(Hsp+DR),p(T) | 186.23 | 3.46 | 0.01 | 0.18 | 5 |
| psi(Hsp+Tcaco+DR+DS),p(T+P) | 186.25 | 3.48 | 0.01 | 0.18 | 8 |
| psi(Tcaco),p(P) | 186.34 | 3.57 | 0.01 | 0.17 | 4 |
| psi(Tcaco),p(E+T+P) | 186.35 | 3.58 | 0.01 | 0.17 | 6 |
| psi(Tcaco),p(E+P) | 186.41 | 3.64 | 0.01 | 0.16 | 5 |
| psi(.),p(.) | 186.58 | 3.81 | 0.01 | 0.15 | 2 |
| psi(Hsp+Tcaco+DS),p(E+T) | 186.62 | 3.85 | 0.01 | 0.15 | 7 |
| psi(WD+Tcaco+DR+DS),p(.) | 186.72 | 3.95 | 0.01 | 0.14 | 6 |
