# Supplementary figures and images for: Occupancy of the Ethiopian endemic Moorland Francolin in pristine and degraded Afroalpine biome using a camera trap approach
Source: Ecol Evol. 2023 Oct 31;13(11):e10551. doi: 10.1002/ece3.10551 (PMC10617016; doi:10.1002/ece3.10551)

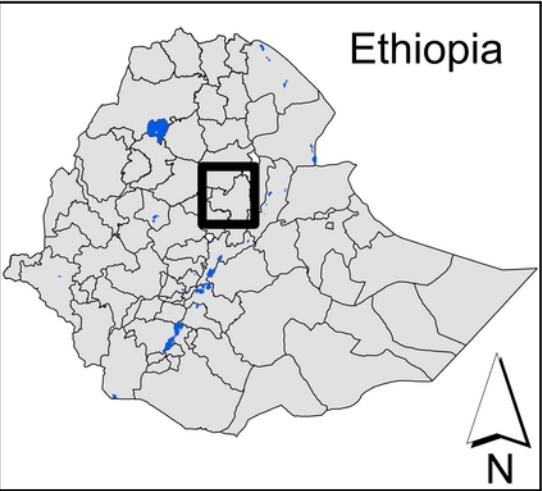

**Legend**

- Sampling Points
- Guasa CFC
- Zonal administration
- Lakes
- Rivers
- Roads

**Altitude (m)**  
High : 4487  
Low : -177

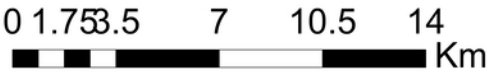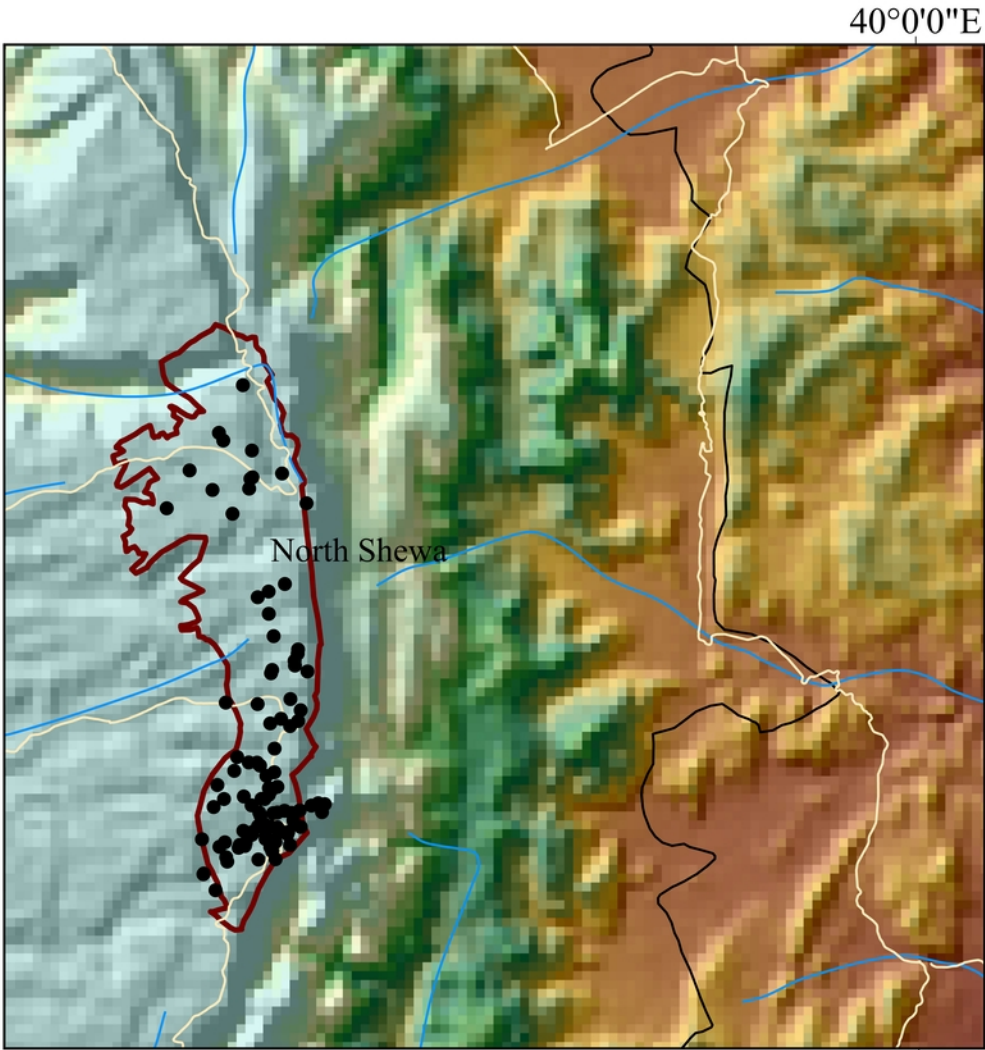

Supplement: Supplementary file 1 — Appendix S1. [file ECE3-13-e10551-s001.zip › ece310551-sup-0002-GCCA_camera_sites.pdf]

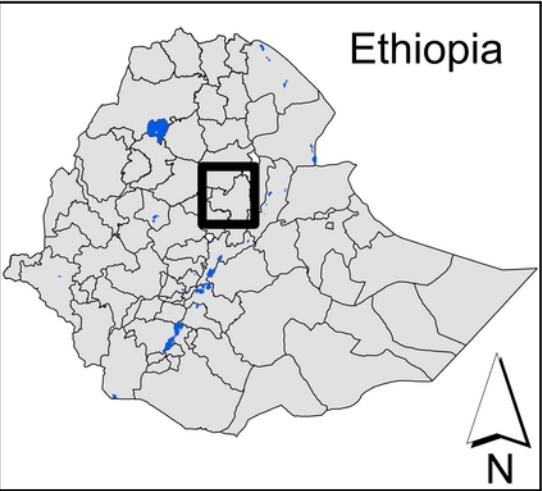

**Legend**

- Sampling Points
- Zonal administration
- Lakes
- Rivers
- Roads
- Altitude (m)**  
High : 4487  
Low : -177

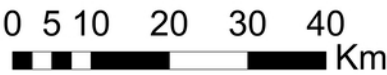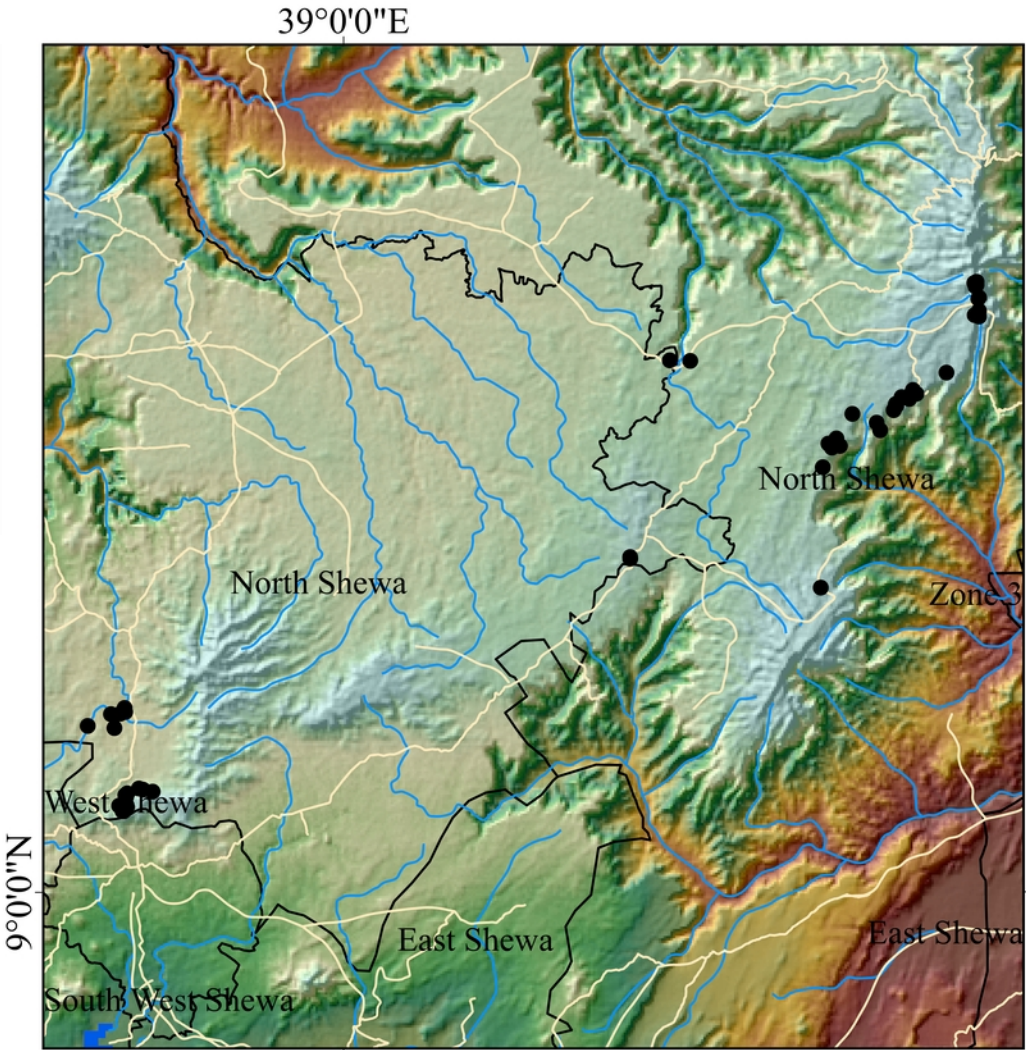

Supplement: Supplementary file 1 — Appendix S1. [file ECE3-13-e10551-s001.zip › ece310551-sup-0003-SEA_camera_sites.pdf]
